# Factors associated with an outbreak of hospital-onset, healthcare facility-associated *Clostridium difficile* infection (HO-HCFA CDI) in a Mexican tertiary care hospital: A case-control study

**DOI:** 10.1371/journal.pone.0198212

**Published:** 2018-05-29

**Authors:** Eric Ochoa-Hein, José Sifuentes-Osornio, Alfredo Ponce de León-Garduño, Pedro Torres-González, Víctor Granados-García, Arturo Galindo-Fraga

**Affiliations:** 1 Department of Hospital Epidemiology, Instituto Nacional de Ciencias Médicas y Nutrición Salvador Zubirán, Mexico City, Mexico; 2 Department of Medicine, Instituto Nacional de Ciencias Médicas y Nutrición Salvador Zubirán, Mexico City, Mexico; 3 Microbiology Laboratory, Instituto Nacional de Ciencias Médicas y Nutrición Salvador Zubirán, Mexico City, Mexico; 4 Epidemiology and Health Services Research Unit, Aging Area, Instituto Mexicano del Seguro Social, Mexico City, Mexico; Cleveland Clinic, UNITED STATES

## Abstract

**Objective:**

To identify clinical and environmental factors associated with an outbreak of hospital-onset, healthcare facility-associated *Clostridium difficile* infection (HO-HCFA CDI).

**Design:**

Case-control study.

**Setting:**

Public, acute care, academic tertiary referral center in Mexico.

**Patients:**

Adults hospitalized ≥48 hours between January 2015 and December 2016 were included. Cases were patients with a first episode of HO-HCFA CDI. Controls were patients with any other diagnosis; they were randomly selected from the hospital discharge database and matched in a 1:2 manner according to the date of diagnosis of case ± 10 days. Variables with p<0.1 were considered for multivariable analysis.

**Results:**

One hundred and fifty-five cases and 310 controls were included. Variables independently associated with HO-HCFA CDI were: exposure to both ciprofloxacin and proton pump inhibitor (PPI) within the last 3 months (OR = 8.07, 95% CI = 1.70–38.16), febrile neutropenia (OR = 4.61, 95% CI = 1.37–15.46), intraabdominal infection (OR = 2.06, 95% CI = 0.95–4.46), referral from other hospitals (OR = 1.99, 95% CI = 0.98–4.05) and an increasing number of antibiotics previously used (OR = 1.28, 95% CI = 1.13–1.46).

**Conclusions:**

Multiple factors were found to be associated with the first episode of HO-HCFA CDI in the setting of an outbreak; of the modifiable risk factors, prior exposure to both ciprofloxacin and PPI was the most important. Referral from other hospitals was an environmental risk factor that deserves further study.

## Introduction

The burden imposed by *Clostridium difficile* infection (CDI) on hospitals and communities is dangerously increasing worldwide [1]. CDI is the most frequent healthcare-associated infection and the main cause of gastroenteritis-associated death in the United States (107,600 hospital-onset infections and 29,000 related deaths in 2011, respectively) [1, 2]. Direct costs related to acute hospital care due to CDI are substantial ($4.8 billion in the United States in 2008) [1].

Although some risk factors for CDI are well established, some differences between study populations have been noted. For instance, although advanced age has been cited as one of the most consistent risk factors for CDI in developed nations, this may not be the case in Latin American countries [3–6]. In our hospital, carbapenems, piperacillin/tazobactam, ceftriaxone and vancomycin are frequently prescribed and are suspected to have a role in CDI risk, despite the fact that clindamycin and quinolones have been the main antibiotics historically associated with CDI. Finally, some reports have shown that the environment plays an important role as a risk factor [7–12], but this has not been confirmed in all settings.

At the beginning of 2015, an outbreak of hospital-onset, healthcare facility-associated CDI (HO-HCFA CDI) was declared in our hospital and it has continued ever since. Therefore, the aim of this study was to identify the clinical and environmental factors associated with HO-HCFA CDI cases in the setting of an outbreak in a tertiary referral hospital in Mexico City.

## Methods

We conducted a case-control study in a public, acute care, academic tertiary care referral hospital. Adult patients hospitalized ≥48 hours in General Wards (167 beds), the Emergency Room (28 beds), the Intensive Care Unit (14 beds), and the Short-Stay Surgery Ward (12 beds) between January 2015 and December 2016 were included; those hospitalized in the Outpatient Parenteral Antimicrobial Therapy rooms and the Postoperative Recovery rooms were not included (9 beds in total). The study was approved by the Institutional Review Board (REF. 1845) and informed consent was waived due to the retrospective nature of the analysis.

Cases were patients who had a first episode of HO-HCFA CDI, which was defined according to the 2010 guidelines [13]: at least three episodes of Bristol chart [14] type 6 or 7 unformed stools in a 24-hour period, acquired after 48 hours of hospitalization and up to hospital discharge. We confirmed the diagnosis by using a two-step algorithm consisting of a positive glutamate-dehydrogenase assay (VIDAS^®^ *C*. *difficile* GDH, Biomérieux, Marcy-l’Étoile, France) followed by a positive A & B toxin-gene amplification test (GeneXpert^®^ *C*. *difficile*/Epi test, Cepheid, Sunnyvale, California, United States). This testing algorithm has been in use in the hospital since 2013 (in previous years, the GDH assay had been followed by the VIDAS^®^ CD A&B toxin detection kit) [15]. All cases were diagnosed by the treating physicians and were registered by the Department of Hospital Epidemiology, according to routine active epidemiological surveillance policies. Patients with a recurrent, indeterminate, community-associated or community-onset, healthcare facility-associated CDI episode as defined by the 2010 guidelines [13] were excluded. Although recurrent CDI cases are one of the most important risk factors for CDI, we decided to exclude them, as we were particularly interested in defining risk factors for a first episode of HO-HCFA CDI in our setting.

Controls were patients with any discharge diagnosis, except ICD-10 code A04.7 (enterocolitis due to *C*. *difficile*). During the study period, the 2014, 2015 and 2016 versions of ICD-10 were used; updating of ICD-10 versions did not affect the case definition that relied on the A04.7 code [16]. All patients with onset of diarrhea in our hospital undergo testing for CDI due to a high prevalence of risk factors for CDI; therefore, those with diarrhea and a negative workup for CDI were also included. Both the medical records and the Hospital Epidemiology database were checked to further verify the absence of any episode of CDI among controls as far back as 3 months before inclusion into the study. Controls were randomly selected from the hospital discharge database with the aid of a simple random number sequence generated in the internet link https://www.randomizer.org. Two controls were matched to each case according to the date of diagnosis of the case ±10 days (which is an arbitrary time period that tried to take into account unknown risk factors possibly related to hospitalization).

Cases and controls whose medical charts were unavailable for consultation were eliminated, but in order to keep the 1:2 matching we used the selection criteria previously defined to replace the eliminated controls with others.

According to information in the Hospital Epidemiology database, it was expected that a room previously occupied by a case would be the factor with the least prevalence in the study population (approximately 19%); therefore, a sample including all cases (n = 161) was deemed to be sufficiently powered to detect an odds ratio of at least 1.92 for the presumed associated factors, assuming a type I error rate of 5%.

The following variables were collected from medical charts: sex, age, economic status, comorbidities, Charlson comorbidity index, date of diagnosis of cases, *C*. *difficile* ribotype for cases (027 or non-027), hospital admission and discharge dates, length of stay (overall length of stay for cases and controls, and length of stay before diagnosis of CDI for cases), and vital status at discharge and at 30 days after discharge. Use of at least one dose of a systemic drug (antibiotics, proton pump inhibitors [PPI], corticosteroids, immunosuppressants and antineoplastic drugs), gastrointestinal surgery, previous hospitalizations, referral from another hospital, and hospitalization in rooms previously occupied by cases were evaluated as far back as 3 months before the date of diagnosis of CDI (cases) or date of hospital discharge (controls). Additionally, rooms occupied by cases and controls during the hospital stay up until the date of diagnosis of CDI (cases) or date of hospital discharge (controls) were registered. We consulted the Hospital Epidemiology database to determine the use of hydrogen peroxide vapor for terminal room disinfection within the last 2 weeks before admission of a case or control to the last occupied bed. *Clostridium difficile-*associated disease pressure values (henceforth referred to as CDI pressure values) were calculated for cases and controls according to previously published methods [17].

In addition to the analysis of the environmental variables previously mentioned, and in an attempt to determine if cross-transmission may have happened, the rooms occupied by cases at the moment of diagnosis, as well as the infecting ribotype (027 or non-027), were visually analyzed by means of a slideshow, which is shown in S1 File.

The following hospital-wide preventive strategies were strengthened upon outbreak detection: a) antibiotic stewardship measures, consisting of automatic alerts, prior authorization and prospective audit and feedback; b) terminal room disinfection with 5,000 ppm of sodium hypochlorite or hydrogen peroxide vapor in rooms occupied by CDI cases (otherwise, 1,000 ppm of sodium hypochlorite was used); c) promotion of hand hygiene in all hospital areas with special emphasis on use of 2% chlorhexidine soap and water after contact with cases; d) strict use of contact precautions on any patient with diarrhea; e) use of 0.5% activated hydrogen peroxide wipes for disinfection of medical instruments in the cohort area (defined below); and f) assignment and disinfection of metal commodes for repeated use by patients in the cohort area.

Adherence to the preventive strategies was only routinely measured for hand hygiene (with either soap and water or alcohol hand rub). This was determined by direct observation of personnel before and after contact with patients, as done routinely during active epidemiological surveillance.

Cohorting of patients with confirmed CDI in the designated area of the second floor was started in March 2015 (see S1 File). This was intended to facilitate adherence to hand hygiene with soap and water due to better availability of sinks and to reduce the influx of visitors and personnel because this area is located at one of the two dead ends of the second floor.

Separately, hospital-wide broad-spectrum antibiotic consumption from 2012 to 2016 was measured using defined daily doses (DDD), according to WHO standards [18]. Briefly, the Pharmacy Department provided the annual number of vials used for each antibiotic: the total annual amount of each antibiotic was first converted to total annual grams and then to annual DDD; the latter was then standardized to 1,000 patient-days for each year. Quinolones (ciprofloxacin and moxifloxacin), clindamycin, carbapenems (ertapenem, imipenem and meropenem), piperacillin/tazobactam, vancomycin and ceftriaxone were included in this analysis.

Statistical analysis was performed with Stata version 14.0 (StataCorp, College Station, TX, USA). Missing values could not be replaced. Absolute and relative frequencies were used to describe categorical variables, and medians and interquartile ranges (IQR) were used to describe non-normally distributed variables. Bivariate analysis of categorical variables was performed with either chi-square test or Fisher’s exact test, as appropriate; two-sample Wilcoxon Rank sum test was used for ordinal and numerical variables. Odds ratios (OR) with their respective 95% confidence intervals (95% CI) and statistical significance were calculated. Variables with p values ≤0.1 were included in multivariable analysis and retained in the model if the p value remained ≤0.1, as has been suggested by another group of authors [19]. Interaction and confounding were intentionally investigated and only statistically significant findings are reported in this paper (interaction terms and interaction analysis can be further consulted in S2 and S3 Files).

## Results

**Fig 1:**
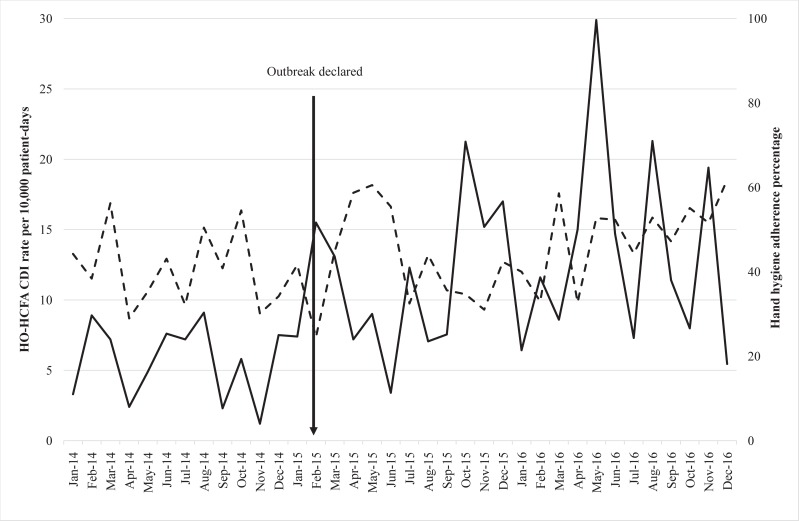
HO-HCFA CDI monthly rates per 10,000 patient-days and hand hygiene monthly adherence rates. Continuous line: *C*. *difficile* infection rates; dashed line: hand hygiene adherence rates (with either soap and water or alcohol hand rub).

During the study period, a median rate of 11.5 cases of HO-HCFA CDI per 10,000 patient-days per month was observed (IQR = 7.4–15.3), as compared with a median rate of 7.0 cases per 10,000 patient-days in 2014 (IQR = 5.4–8.0), p = 0.002 (Fig 1). The mean hand hygiene adherence rate across the study period was 43.8±10.4% (Fig 1).

A total of 329 CDI cases were confirmed during the study period. Forty-eight recurrent cases (14.6%), 13 indeterminate cases (3.9%), 37 community-associated cases (11.2%), and 70 community-onset, healthcare facility-associated cases (21.3%) were excluded; after elimination of 6 HO-HCFA CDI cases due to unavailable medical charts, a total of 155 cases were included for analysis. Of 310 controls selected, 14 were eliminated due to unavailable medical charts and were randomly replaced with others.

Cases and controls had similar sex and economic status distributions (Table 1). There were no differences in age between cases and controls; 42 cases (27.1%) and 89 controls (28.7%) were ≥65 years old (OR = 0.92, 95% CI = 0.59–1.42, p = 0.718). The Emergency Room admitted 90 out of 127 cases (70.9%) and 156 out of 243 controls (64.2%), p = 0.197. More cases than controls were hospitalized on the second floor before diagnosis of CDI: 13 out of 86 cases (15.1%) and 10 out of 160 controls (6.3%), p = 0.022. No other differences in spatial distribution within the hospital between cases and controls were found.

**Table 1:** Bivariate analysis of demographic, clinical and environmental variables.

Variable	Cases^a^ (n = 155)	Controls^a^ (n = 310)	OR	95% CI	p
Female	84 (54.2)	170 (54.8)	0.97	0.66–1.43	0.895
Age^b^	55 (38–66)	52 (37–68)	1.00	0.99–1.01	0.883
Economic status^c^	2 (2–3)	2 (2–3)	1.02	0.90–1.16	0.491
Charlson comorbidity index	4 (2–6)	4 (2–6)	0.99	0.92–1.06	0.804
Total length of stay^b^	25 (17.5–40)	10 (6–18)	—	—	<0.001
Length of stay before diagnosis^b^^,^ ^d^	13 (7.8–20)	10 (6–18)	1.01	0.99–1.02	0.077
All-cause death rate upon discharge	10/154 (6.5)	25/309 (8.1)	0.78	0.36–1.68	0.540
All-cause 30-day death rate	12/149 (8.1)	31/287 (10.8)	0.72	0.36–1.45	0.361
Febrile neutropenia	14/148 (9.5)	5/297 (1.7)	6.10	2.15–17.28	<0.001
Leukemia	14/148 (9.5)	14/297 (4.7)	2.11	0.97–4.55	0.052
Lymphoma	12/148 (8.1)	13/297 (4.4)	1.92	0.85–4.33	0.107
Previous gastrointestinal surgery	54/147 (36.7)	77/292 (26.4)	1.62	1.06–2.47	0.025
Intraabdominal infection	25/148 (16.9)	18/297 (6.1)	3.15	1.65–5.98	<0.001
Gastrointestinal tract infection	25/148 (16.9)	36/297 (12.1)	1.47	0.84–2.56	0.168
Non-gastrointestinal tract infection	79/148 (53.4)	124/297 (41.8)	1.59	1.07–2.37	0.020
Gastrointestinal tract ostomy	7/148 (4.7)	12/297 (4.0)	1.17	0.45–3.06	0.735
Liver transplantation	4/148 (2.7)	8/297 (2.7)	1.0	0.29–3.38	1.0
Kidney transplantation	3/148 (2.0)	20/297 (6.7)	0.28	0.08–0.98	0.035
Autoimmune disease	12/148 (8.1)	40/297 (13.5)	0.56	0.28–1.11	0.097
Antineoplastic drugs	29/147 (19.7)	37/283 (13.1)	1.63	0.95–2.78	0.069
Systemic steroids	60/141 (42.6)	117/277 (42.2)	1.01	0.67–1.52	0.951
Immunosuppressants	27/145 (18.6)	56/278 (20.1)	0.90	0.54–1.51	0.708
Proton pump inhibitors	89/134 (66.4)	144/272 (52.9)	1.75	1.14–2.70	0.010
Enteral nutrition	18/133 (13.5)	23/267 (8.6)	1.66	0.86–3.19	0.126
Previous antibiotic use	141/147 (95.9)	230/288 (79.9)^e^	5.92	2.49–14.09	<0.001
Number of antibiotics previously used^b^	3 (2–5)	2 (1–3)	1.41	1.25–1.58	<0.001
Any quinolone	25/147 (17.0)	23/275 (8.4)	2.24	1.22–4.11	0.008
Ciprofloxacin	16/147 (10.9)	6/275 (2.2)	5.47	1.97–17.41	<0.001
Moxifloxacin	3/147 (2.0)	11/275 (4.0)	0.5	0.09–1.93	0.396
Levofloxacin	7/147 (4.8)	6/275 (2.2)	2.24	0.63–8.22	0.151
Meropenem or imipenem	66/147 (44.9)	74/275 (26.9)	2.21	1.45–3.36	<0.001
Ertapenem	53/147 (36.1)	64/275 (23.3)	1.85	1.20–2.88	0.005
Any carbapenem	94/147 (63.9)	118/275 (42.9)	2.35	1.53–3.64	<0.001
Vancomycin	77/147 (52.4)	98/275 (35.6)	1.98	1.32–2.98	0.001
Piperacillin/ tazobactam	56/147 (38.1)	71/275 (25.8)	1.76	1.15–2.71	0.009
Ceftriaxone	34/147 (23.1)	44/275 (16.0)	1.58	0.95–2.60	0.072
Ceftazidime	5/147 (3.4)	2/275 (0.7)	4.80	0.92–25.08	0.053
Ceftriaxone or ceftazidime	39/147 (26.5)	45/275 (16.4)^f^	1.84	1.13–3.0	0.013
Clindamycin	5/147 (3.4)	7/275 (2.5)	1.34	0.42–4.32	0.760
Duration of previous antibiotic use^b^	12 (7–23)	3 (0–10)	1.03	1.01–1.05	0.003
Previous hospitalization	106/147 (72.1)	149/281 (53.0)	2.29	1.49–3.52	<0.001
Previous stay in our hospital	64/147 (43.5)	113/281 (40.2)	1.14	0.76–1.71	0.507
Referral from another hospital	25/147 (17.0)	24/281 (8.5)	2.19	1.20–3.99	0.009
Referral from another hospital and previous stay in our hospital	17/147 (11.6)	12/281 (4.3)	2.93	1.36–6.31	0.004
Room previously used by case	26/92 (28.3)	37/191 (19.4)	1.64	0.91–2.92	0.092
Hydrogen peroxide vapor disinfection	4/94 (4.3)	10/193 (5.2)	0.81	0.24–2.66	1.0

HO-HCFA CDI, hospital-onset, healthcare facility-associated *Clostridium difficile* infection; OR, odds ratio; CI, confidence interval.

^a^ Absolute frequency (%), unless otherwise stated.

^b^ Median (interquartile range).

^c^ The scale used in the National Health Institutes in Mexico is composed of 6 levels, each one reflecting a different discount rate applied to the hospital discharge bill, as follows: 1 = 96%, 2 = 84%, 3 = 64%, 4 = 43%, 5 = 23%, 6 = 0%.

^d^ Length of stay before diagnosis of CDI (cases) or hospital discharge date (controls).

^e^ Specific antibiotic used for preoperative antibiotic prophylaxis was not mentioned in the charts of 13 control patients.

^f^ One patient used both ceftriaxone and ceftazidime.

Total length of stay (but not length of stay before diagnosis of CDI) was significantly longer for cases. In-hospital and 30-day mortality rates were similar between cases and controls.

Infecting ribotype information was available for 149 cases; ribotype 027 was identified in 68 cases (45.6%) and non-027 ribotype in 81 cases (54.4%). Exposure to quinolones, ciprofloxacin, moxifloxacin and levofloxacin was stratified by ribotype but no difference was revealed between cases infected with 027 and non-027 strains.

As shown in Table 1, febrile neutropenia, previous gastrointestinal surgery, intraabdominal infection, non-gastrointestinal tract infection (i.e., infection in any organ system excluding the gastrointestinal tract), previous PPI use, previous antibiotic use, previous hospitalization and referral from another hospital were more frequent in cases. Kidney transplantation was more frequent in controls. An interaction between ciprofloxacin and PPI (hereafter referred to as “exposure to both ciprofloxacin and PPI”) was found (stratified analysis is shown in Table 2), but not for PPI and other quinolones or for PPI and other antibiotics. After multivariable analysis, exposure to both ciprofloxacin and PPI, febrile neutropenia, intraabdominal infection, referral from another hospital and an increasing number of antibiotics used during the hospital stay remained in our model (Table 3).

**Table 2:** Stratified analysis for concomitant use of PPIs and quinolones (ciprofloxacin, moxifloxacin and levofloxacin).

		Cases^a^	Controls^a^	OR	95% CI	p
Quinolone exposure	PPI users	20/88 (22.7)	16/139 (11.5)	2.26	1.03–4.98	0.024
	PPI non-users	3/43 (7.0)	6/115 (5.2)	1.36	0.21–6.73	0.704
Exposure to both quinolone and PPI^b^		20/131 (15.3)	16/254 (6.3)	2.68	1.26–5.74	0.004
Ciprofloxacin exposure^c^	PPI users	13/88 (14.8)	2/139 (1.4)	11.87	2.55–110.02	<0.001
	PPI non-users	1/43 (2.3)	4/115 (3.5)	0.66	0.01–6.94	1.0
Exposure to both ciprofloxacin and PPI^b^		13/131 (9.9)	2/254 (0.8)	13.88	3.05–127.79	<0.001
Moxifloxacin exposure	PPI users	2/88 (2.3)	9/139 (6.5)	0.33	0.03–1.68	0.209
	PPI non-users	1/43 (2.3)	1/115 (0.01)	2.71	0.03–214.88	0.471
Exposure to both moxifloxacin and PPI^b^		2/131 (1.5)	9/254 (3.5)	0.42	0.04–2.08	0.345
Levofloxacin exposure	PPI users	6/88 (6.8)	5/139 (3.6)	1.96	0.48–8.36	0.344
	PPI non-users	1/43 (2.3)	1/115 (0.01)	2.71	0.03–214.88	0.471
Exposure to both levofloxacin and PPI^b^		6/131 (4.6)	5/254 (1.9)	2.39	0.59–10.08	0.195

PPI, proton pump inhibitor; OR, odds ratio; CI, confidence interval.

^a^ Absolute frequency (%).

^b^ Exposure to both PPI and quinolone occurred within 3 months before diagnosis of CDI (cases) or the date of hospital discharge (controls). Non-overlapping exposures may have occurred.

^c^ Mantel-Haenszel test of homogeneity was statistically significant (p = 0.035); therefore, an interaction exists between ciprofloxacin and PPI.

**Table 3:** Factors associated with HO-HCFA CDI in logistic regression analysis.

Variable	aOR	95% CI	Coefficient	p
Exposure to both ciprofloxacin and PPI	8.07	1.70–38.16	2.09	0.008
Febrile neutropenia	4.61	1.37–15.46	1.53	0.013
Intraabdominal infection	2.06	0.95–4.46	0.73	0.064
Referral from other hospital	1.99	0.98–4.05	0.69	0.056
Increasing number of antibiotics previously used	1.28^a^	1.13–1.46	0.25	<0.001

Constant for the model = 0.20 (95% CI = 0.13–0.30).

HO-HCFA CDI, hospital onset, healthcare facility-associated *Clostridium difficile* infection; aOR, adjusted odds ratio; CI, confidence interval; PPI, proton pump inhibitor.

^a^ aOR for every increase in the number of antibiotics previously used.

Median CDI pressure values for cases and controls were 1.74 (IQR = 0.96–5.41) and 1.58 (IQR = 0.71–4.67), respectively (p = 0.539).

**Fig 2:**
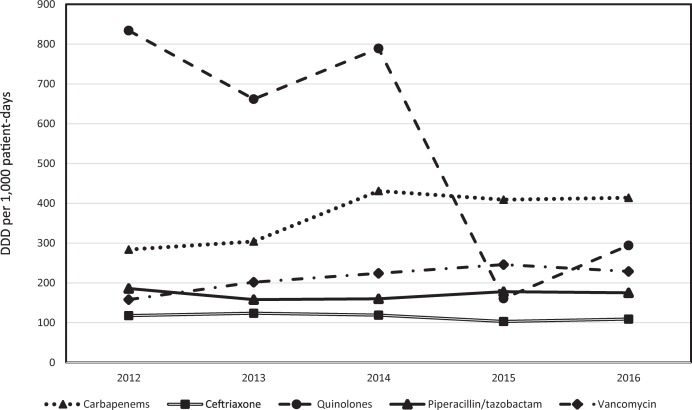
Antibiotic consumption (DDD per 1,000 patient-days per year, 2012–2016).

Analysis of hospital-wide broad-spectrum antibiotic consumption from 2012 to 2016 revealed a mixed trend for quinolones (reduction from 834 DDD in 2012 to 161 DDD in 2015, but an increase to 294 DDD in 2016 that coincided with the outbreak), a slight increasing trend for carbapenems and vancomycin, and a steady trend for piperacillin/tazobactam and ceftriaxone. Detailed results are shown in Fig 2. Clindamycin consumption was below 10 DDD per 1,000 patient-days for any given year and therefore, was not plotted.

## Discussion

This study showed that the main factors associated with the first episode of HO-HCFA CDI in the setting of an outbreak in our hospital were exposure to both ciprofloxacin and PPI, febrile neutropenia, an increase in the number of antibiotics used before the CDI episode, intraabdominal infection and referral from other hospitals. Although the former two variables had p values above 0.05, they fulfilled our predefined statistical criteria (p<0.1) and are also biologically plausible and significant.

In our setting, previous exposure to systemic antibiotics is a major factor associated with HO-HCFA CDI and risk varies by antibiotic class, as has been demonstrated by others [20–24]. In particular, ciprofloxacin showed the greatest association in combination with PPI, as has been described previously [25]. One postulated mechanism is that the PPI may alter the absorption of antibiotics, resulting in a greater concentration of the latter in the intestinal lumen [26]; however, this hypothesis has not been confirmed. As stated by others [20–27], we found that carbapenems, third generation cephalosporins, piperacillin/tazobactam and vancomycin were associated with HO-HCFA CDI, although only in bivariate analysis. Finally, the association between an increasing number of antibiotics previously used and CDI was described in a previous study done in our hospital and was confirmed in the present one [3].

The analysis of hospital-wide broad-spectrum antibiotic use revealed a steep increase in quinolone consumption between 2015 and 2016 after a declining tendency in previous years (as opposed to declining or stable tendencies for the other antibiotics). Although renewed efforts to reduce quinolone consumption in the hospital are underway and are undoubtedly needed, the majority of patients treated with quinolones had received at least one dose before being admitted to our hospital; therefore, efforts in our hospital should also be paralleled by efforts at the community level to achieve a reduction of cases of CDI. [28]

Some differences between our study and others were noted. Increasing age is one of the most consistently cited risk factors for CDI, but our study, in agreement with other reports [3–6], did not find such an association. Differences in CDI pressure values between cases and controls were not reproduced in our study due to the fact that the value for controls in our study was substantially higher than that reported elsewhere [17]. Great efforts were devoted to discharge stable and uncomplicated CDI cases for ambulatory treatment as early as possible in an attempt to reduce CDI pressure values, but many patients remained hospitalized due to other comorbidities. Although CDI pressure values for cases and controls were similar in our study, the appearance of new HO-HCFA CDI cases in close spatial and temporal proximity to others suggests cross-transmission may have occurred (see S1 File). In our consideration, this graphical analysis is a way of depicting the influence the environment has had on this outbreak, despite the fact that statistical results do not fully support this observation.

The fact that referral from another hospital was associated with HO-HCFA CDI may be a plausible explanation for outbreak persistence despite commonplace infection control measures in our hospital. One hypothesis is that referral from another hospital is a marker of environmental exposure to *C*. *difficile*; this hypothesis is supported by previous studies that showed that CDI rates in neighboring referral hospitals were correlated with those of hospitals from which the patients were transferred [11, 12]. In fact, screening of high-risk patients upon admission (including those with previous hospital admissions) has been recently suggested as an adjunct measure for control of CDI in hospitals [29].

Our study has strengths. This is an updated report of factors associated with CDI in the setting of an outbreak in a Mexican hospital. It included a broad range of hospitalized patients with different comorbidities and information about many associated factors and confounders, better reflecting the real scenario in our hospital. It has also shown that some associated factors could be particular to our hospital, a fact that better emphasizes the need to perform research in different clinical settings [30]. Additionally, the results may help to develop and validate a predictive score to identify patients at risk for HO-HCFA CDI upon admission to our hospital, in an attempt to shift the prevailing prevention paradigm from a secondary prevention strategy to a primary prevention one.

We acknowledge limitations. Missing data could not be recovered for many environmental variables not registered in charts. We could not analyze the impact of the preventive strategies as a whole on HO-HCFA CDI rates since we were only able to measure adherence to hand hygiene and hydrogen peroxide vapor use. Differentiation of adherence to hand hygiene with soap and water or alcohol hand rub was not possible, although a separate surrogate analysis is offered (S4 File); despite concerns that the alcohol hand rub is not able to eliminate spores, its use is nonetheless not contraindicated in hospital outbreaks of CDI, as stated in the updated 2017 guidelines [31]. Molecular analyses of *C*. *difficile* strains isolated during the outbreak are still pending and could undoubtedly aid in the interpretation of transmission dynamics.

## Conclusions

The factors found to be independently associated with the first episode of HO-HCFA CDI during an outbreak in a tertiary referral hospital in Mexico were exposure to both ciprofloxacin and PPI, febrile neutropenia, intraabdominal infection, referral from another hospital and an increasing number of antibiotics previously used. Patients referred to us from other hospitals could be asymptomatic carriers of *C*. *difficile*, and this might be an interesting research subject in Mexico.

## Supporting information

S1 File Graphical representation of temporal and spatial evolution of the outbreak within the hospital. (PDF)

S2 File Database. (XLSX)

S3 File Interaction analysis and simplified model-building strategy. (PDF)

S4 File Audit of chlorhexidine soap and alcohol hand rub consumption, 2015–2016. (PDF)
