# Supplementary material for: Factors associated with an outbreak of hospital-onset, healthcare facility-associated Clostridium difficile infection (HO-HCFA CDI) in a Mexican tertiary care hospital: A case-control study
Source: PLoS One. 2018 May 29;13(5):e0198212. doi: 10.1371/journal.pone.0198212 (PMC5973614; doi:10.1371/journal.pone.0198212)
Supplement: S1 File — (PDF) [file pone.0198212.s001.pdf]

Supporting information

Graphical representation of temporal and spatial evolution of the outbreak within the hospital

Spatial distribution within the hospital

- Low level: Critical Care Unit, Emergency Room, Operating Rooms.
  - Emergency Room not represented due to remodelling during 2015-2016.
  - No cases diagnosed in Operating Rooms; therefore, not represented.
- First, second, third and fourth floors: hospitalization wards.
  - *Clostridium difficile* cohorting area located in second floor.

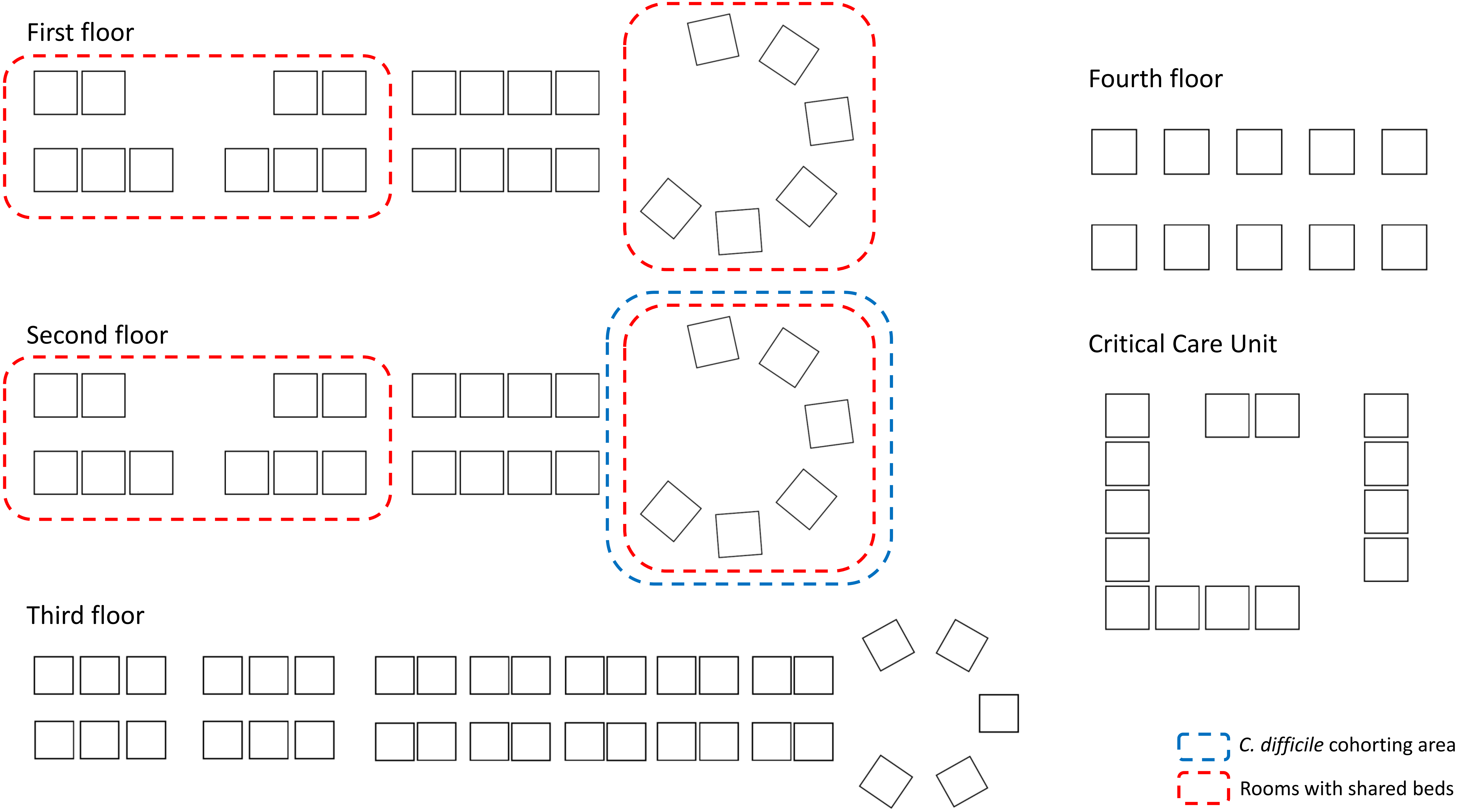

First floor
Second floor
Third floor
Fourth floor
Critical Care Unit
C. difficile cohorting area
Rooms with shared beds

Graphical schema of spatial distribution

- Each square symbolizes either a shared room (2- or 4-patient rooms) or an individual room.

- First and second floors: 16 shared rooms and 8 individual rooms in each.
- Third floor: 37 individual rooms.
- Fourth floor: 10 individual rooms.
- Critical Care Unit: 14 individual rooms.

Symbology

- Each squared dot represents a new patient diagnosed with hospital-onset, healthcare facility-associated *C. difficile*-associated infection (HO-HCFA CDI).
  - Red dots: 027 *Clostridium difficile* strain.
  - Green dots: Non-027 *Clostridium difficile* strains.

- Temporal analysis:
  - Each month includes new cases identified as well as accumulated cases from previous months to better visualize affected areas.

- Dashed lines encircle rooms where different new cases occurred in close proximity to each other with less than one month of difference.

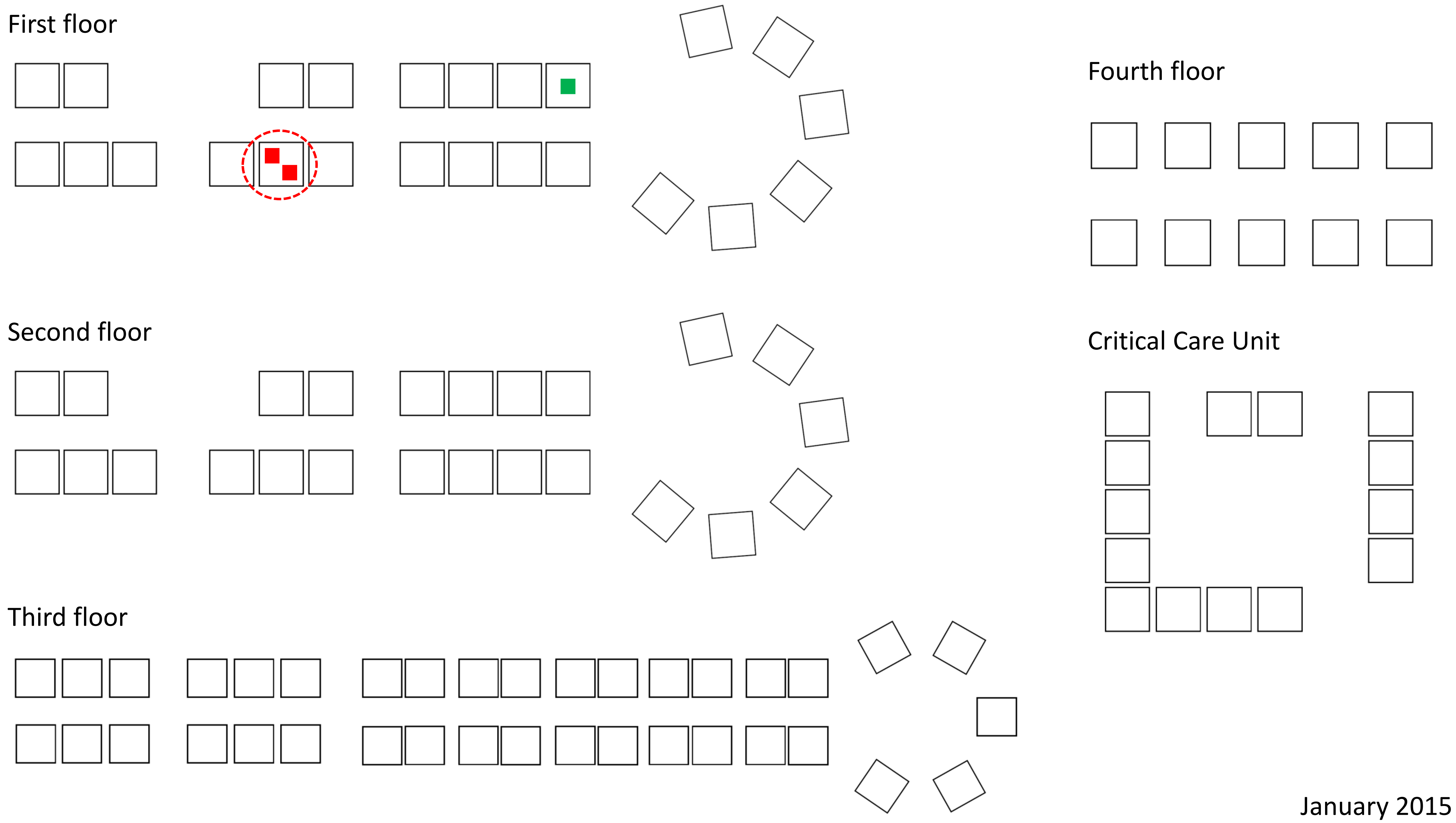
First floor
Fourth floor
Second floor
Critical Care Unit
Third floor
January 2015

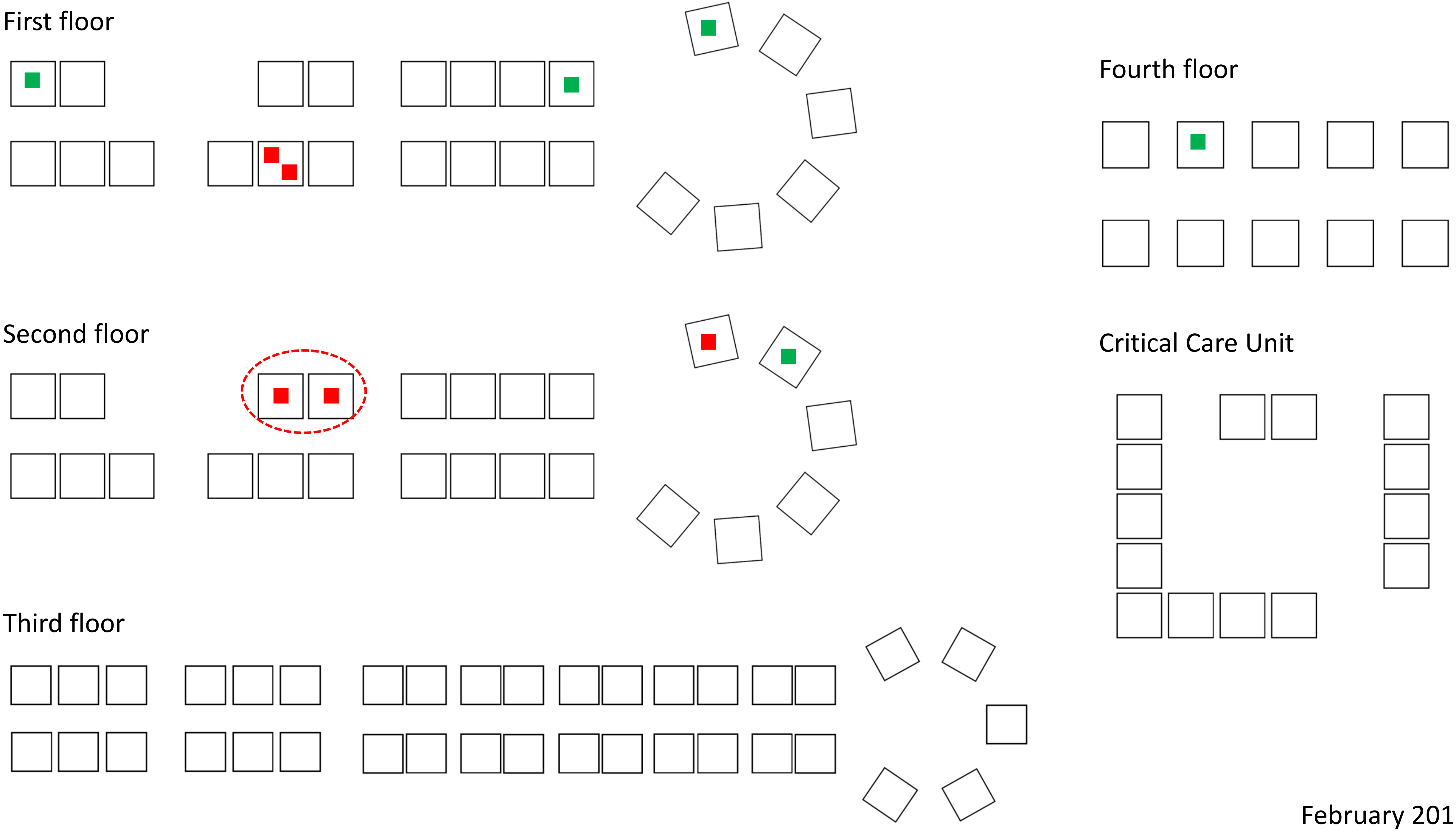

February 2015

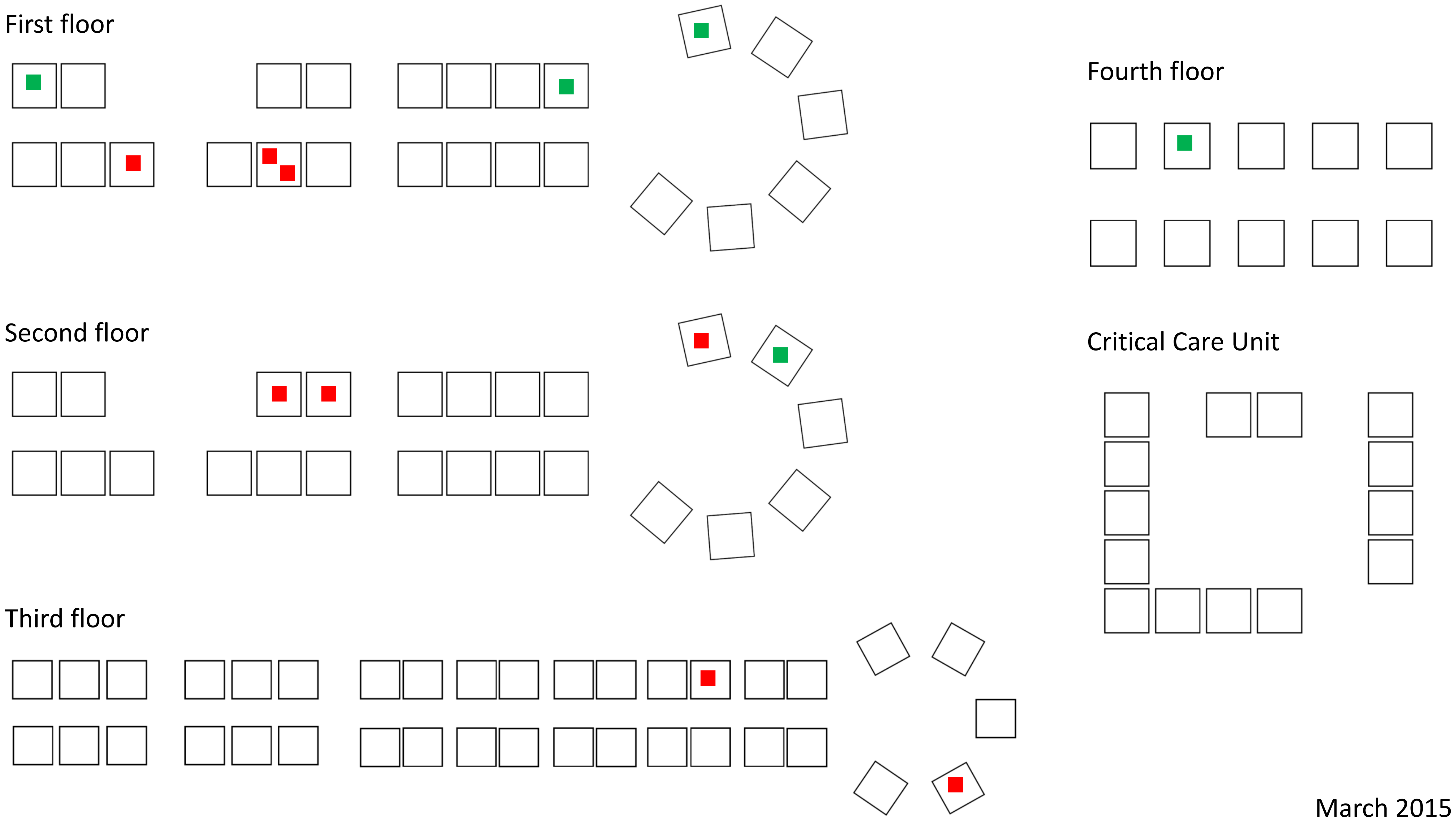
First floor
Fourth floor
Second floor
Critical Care Unit
Third floor
March 2015

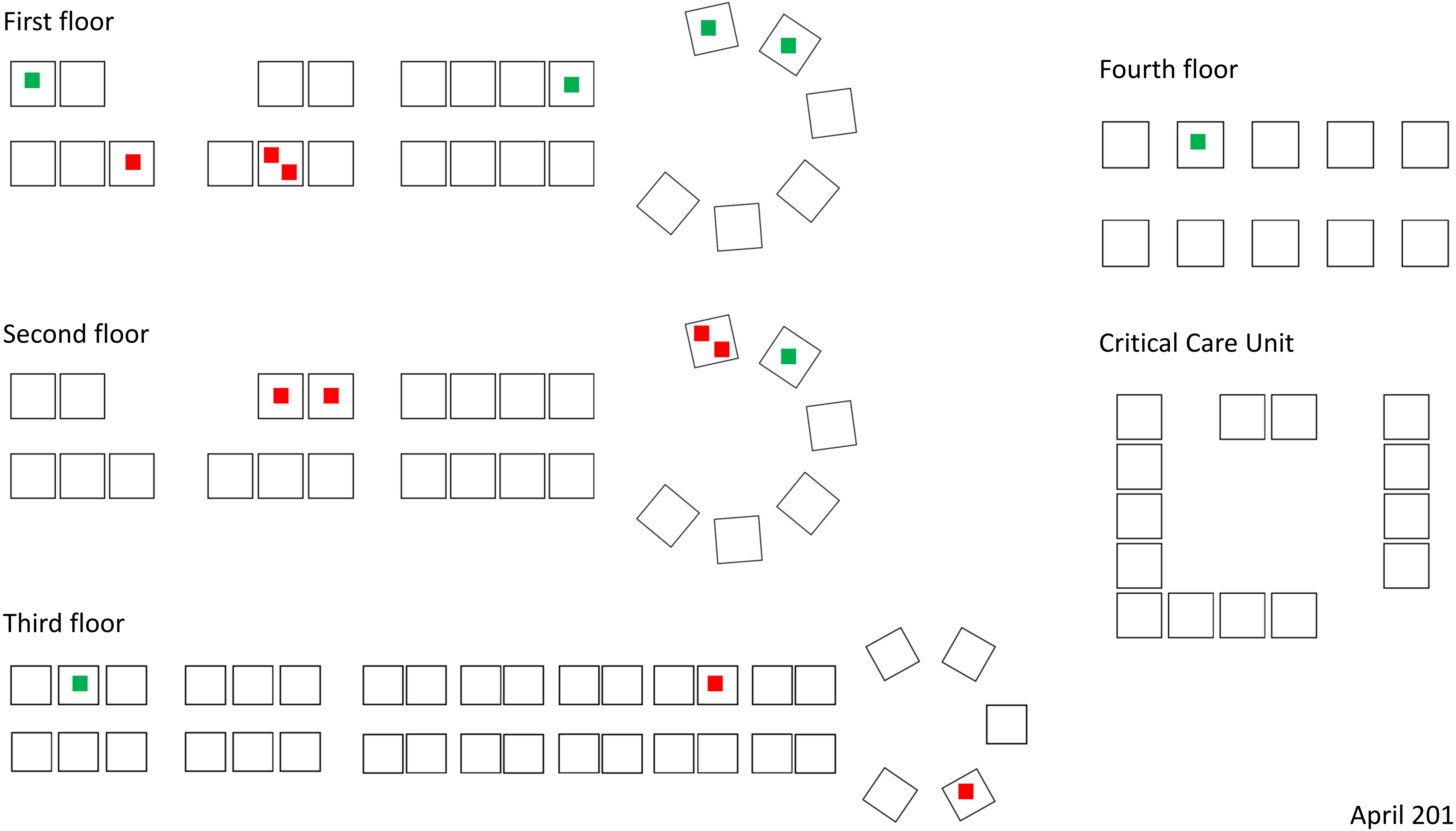
First floor
Second floor
Third floor
Fourth floor
Critical Care Unit

April 2015

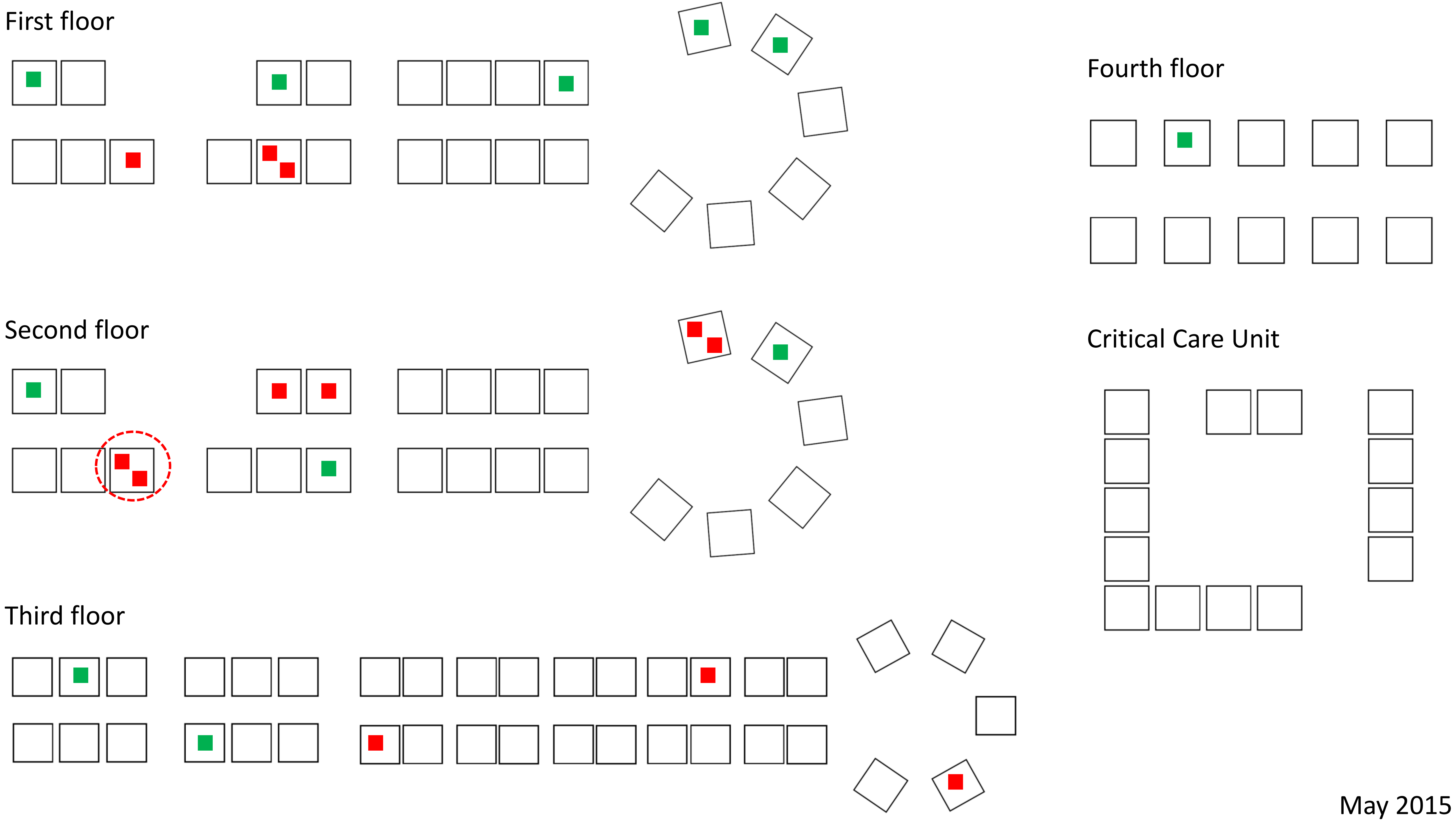
First floor
Second floor
Third floor
Fourth floor
Critical Care Unit
May 2015

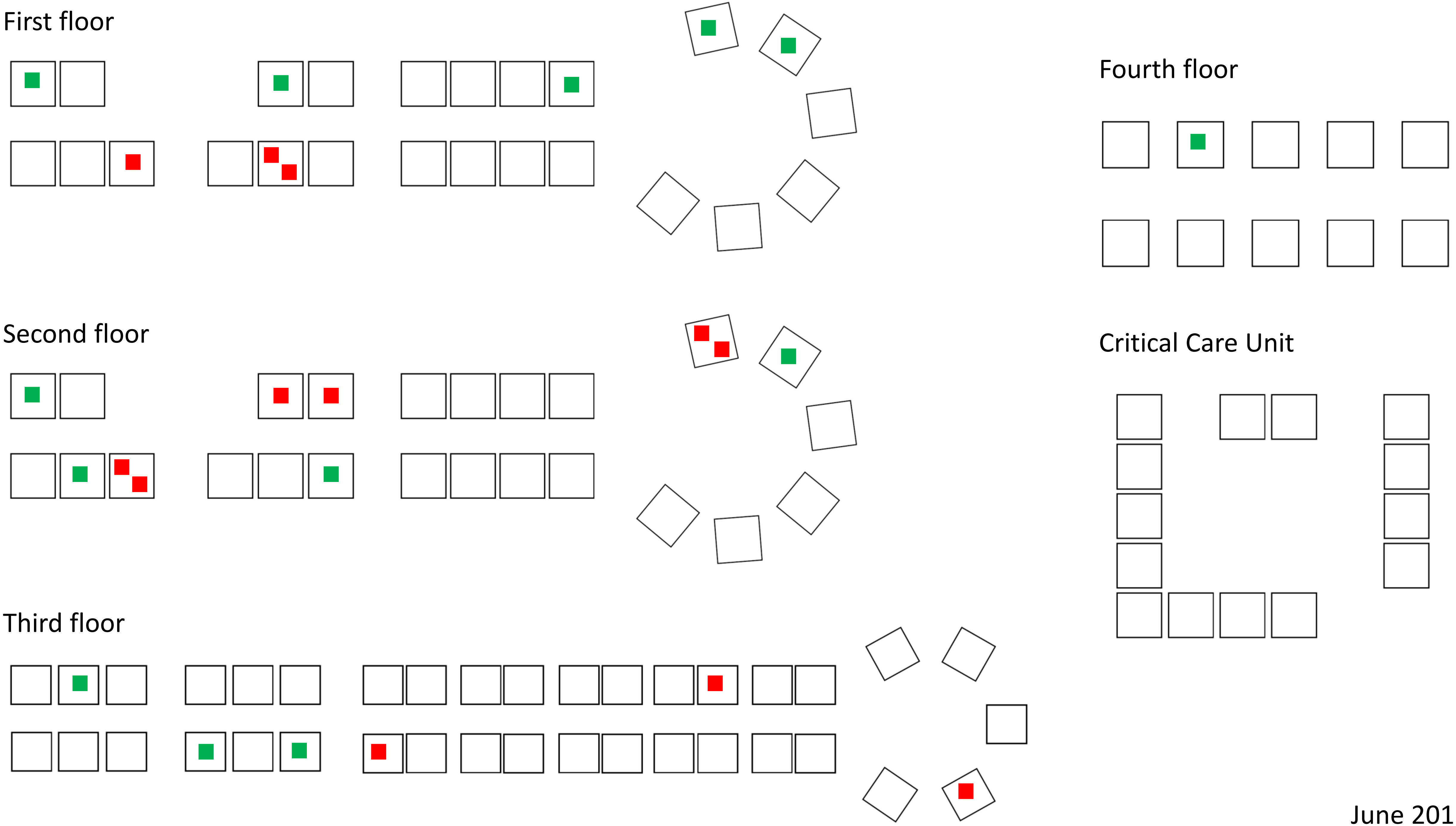
First floor
Second floor
Third floor
Fourth floor
Critical Care Unit

June 2015

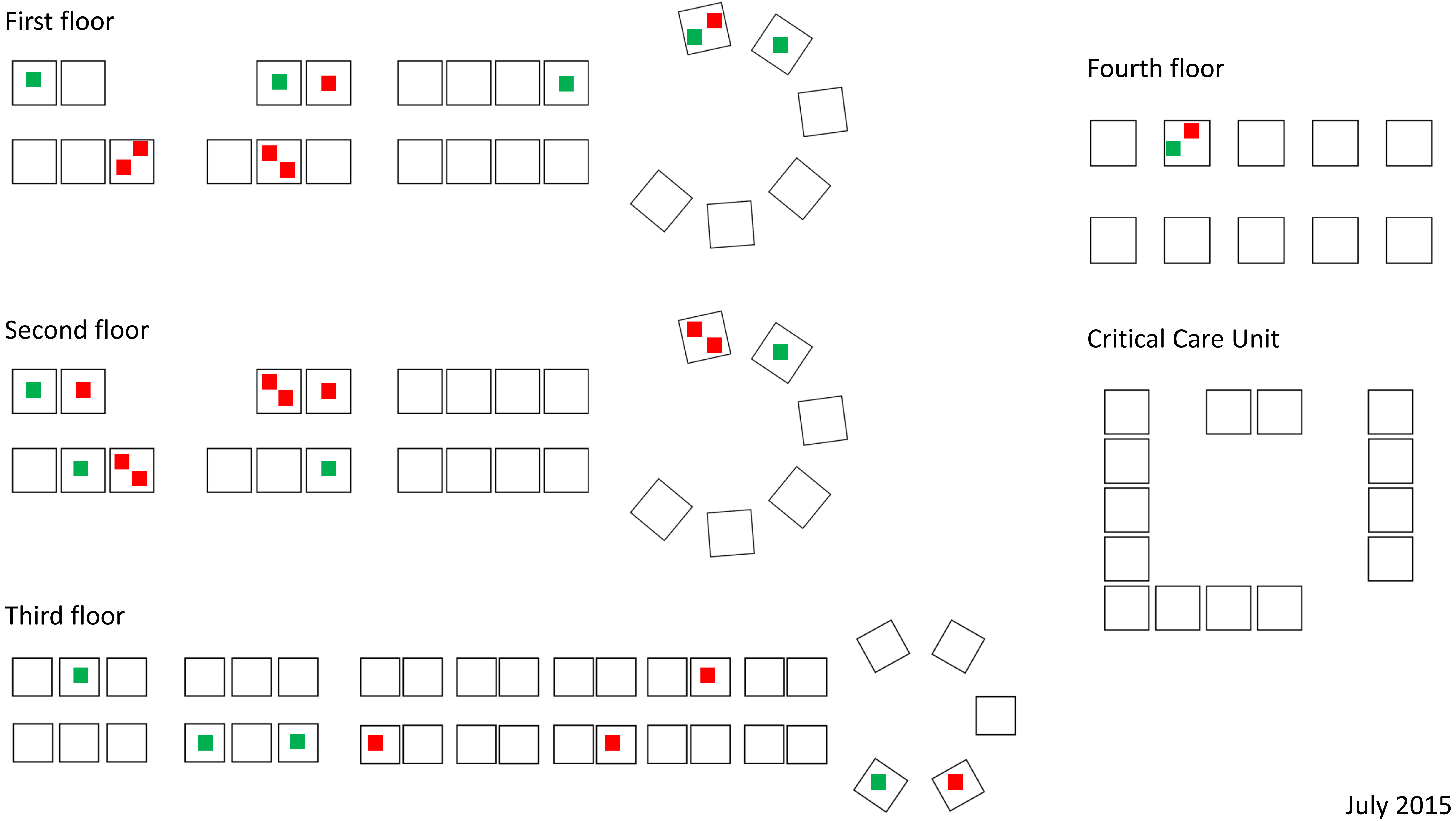
First floor
Fourth floor
Second floor
Critical Care Unit
Third floor
July 2015

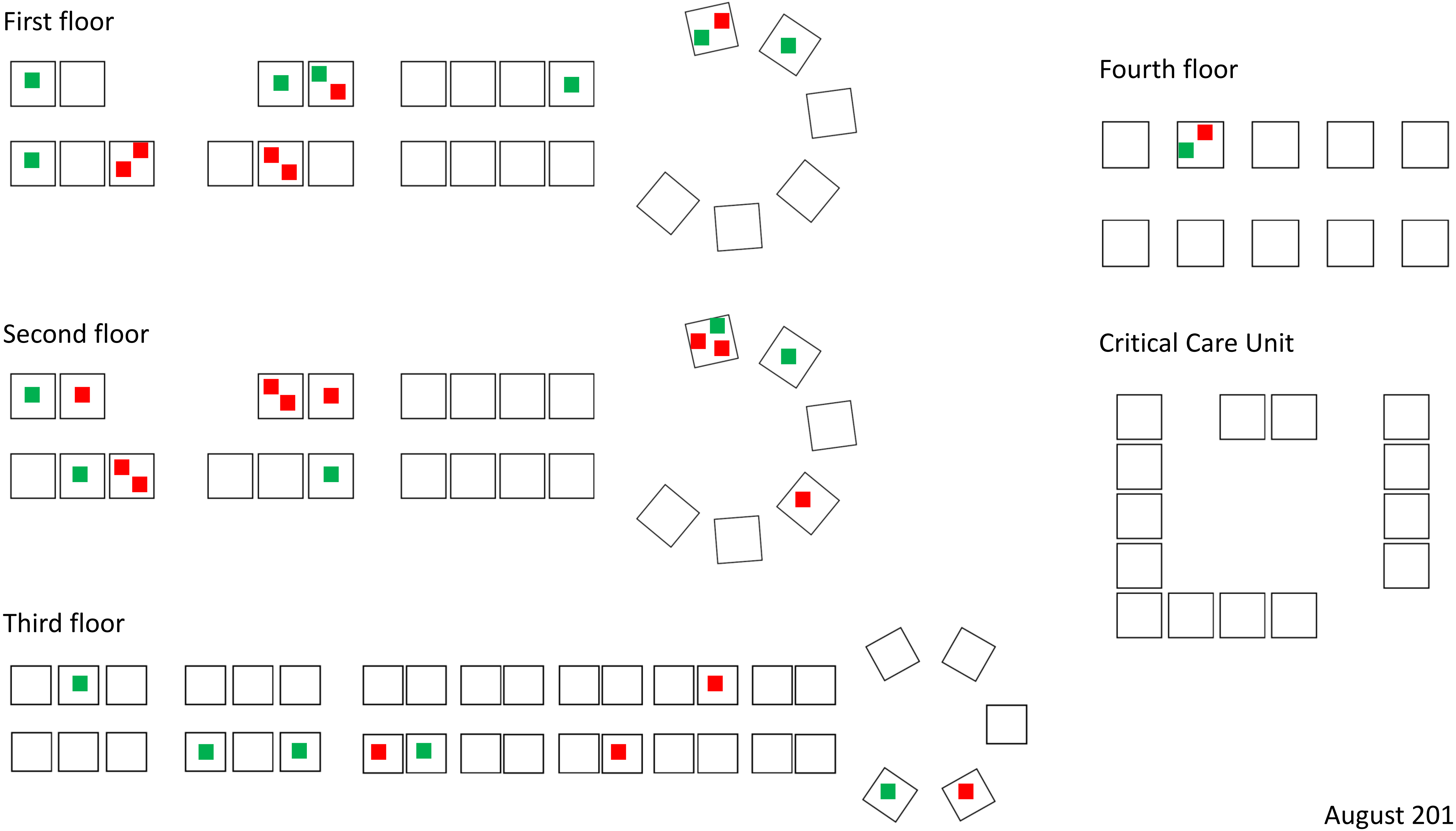
First floor
Second floor
Third floor
Fourth floor
Critical Care Unit

August 2015

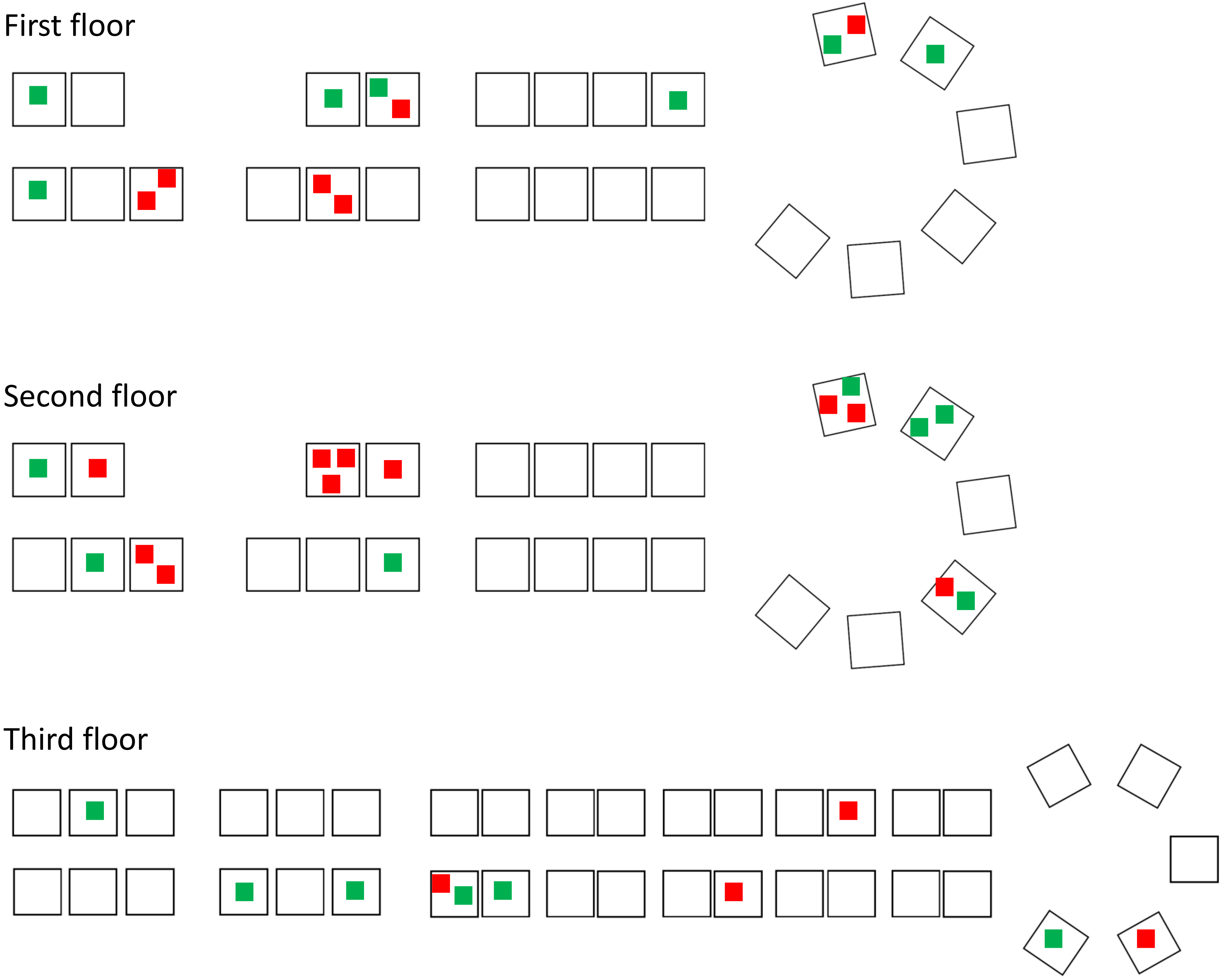

September 2015

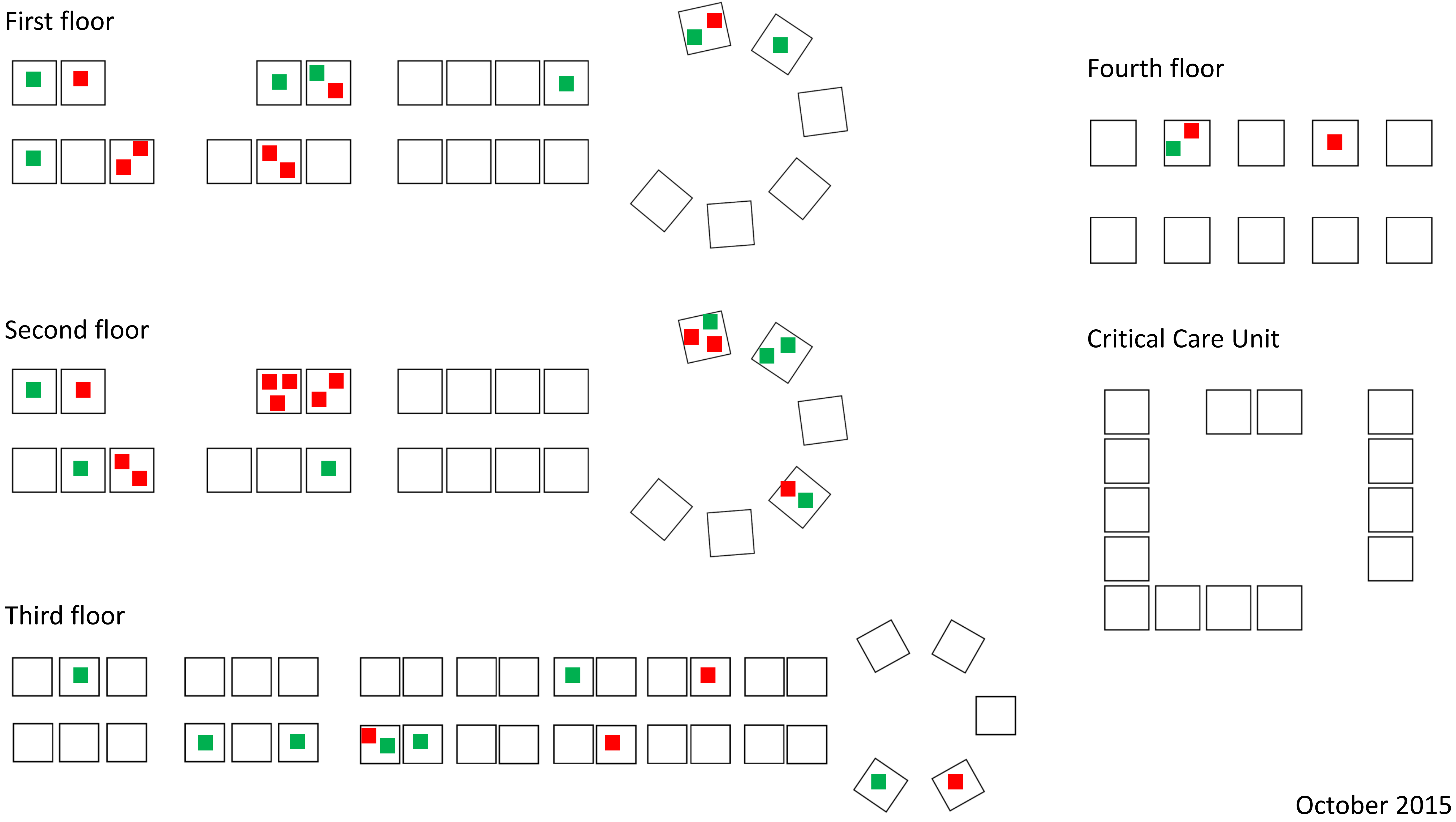
First floor
Fourth floor
Second floor
Critical Care Unit
Third floor
October 2015

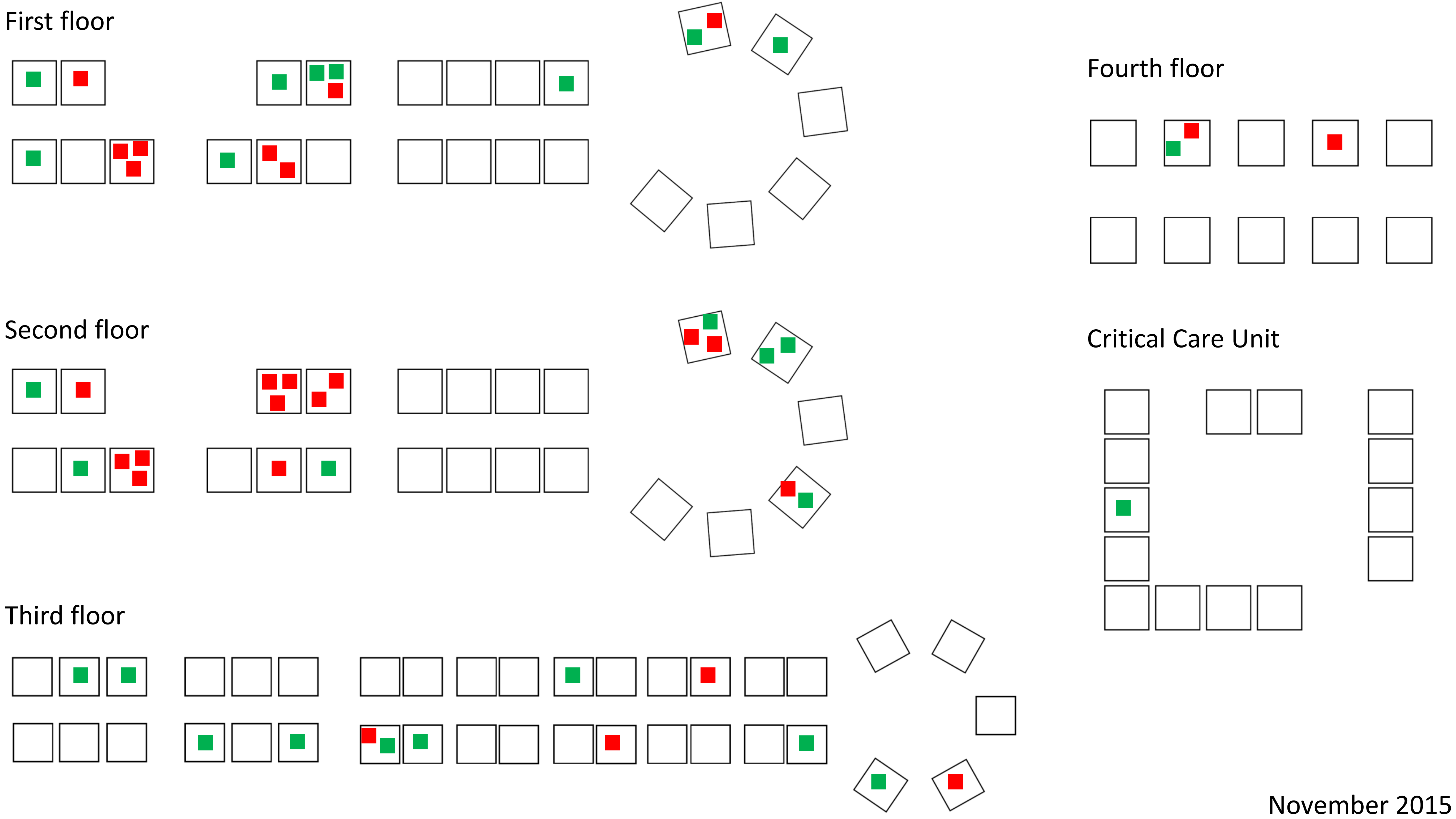
First floor
Fourth floor
Second floor
Critical Care Unit
Third floor
November 2015

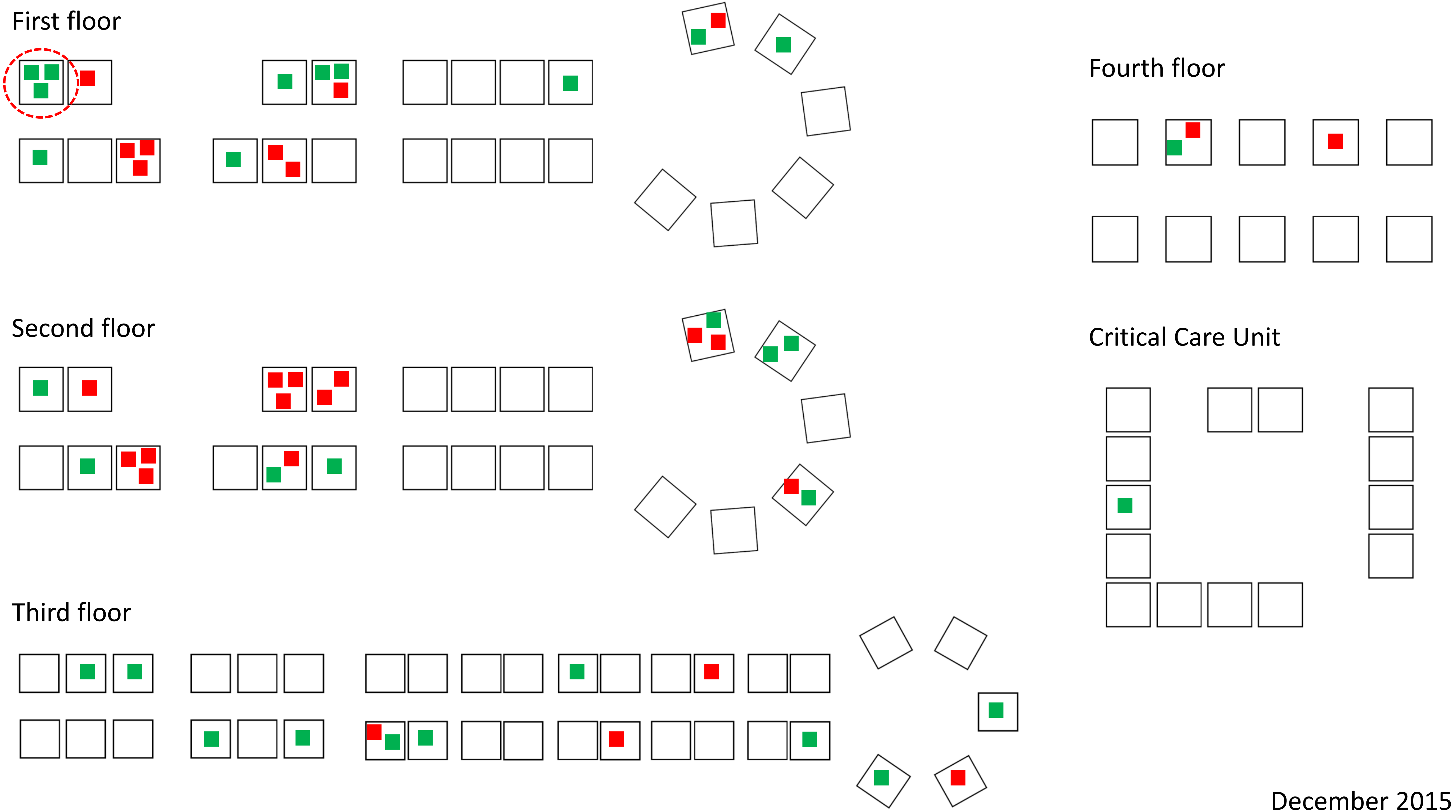
First floor
Fourth floor
Second floor
Critical Care Unit
Third floor
December 2015

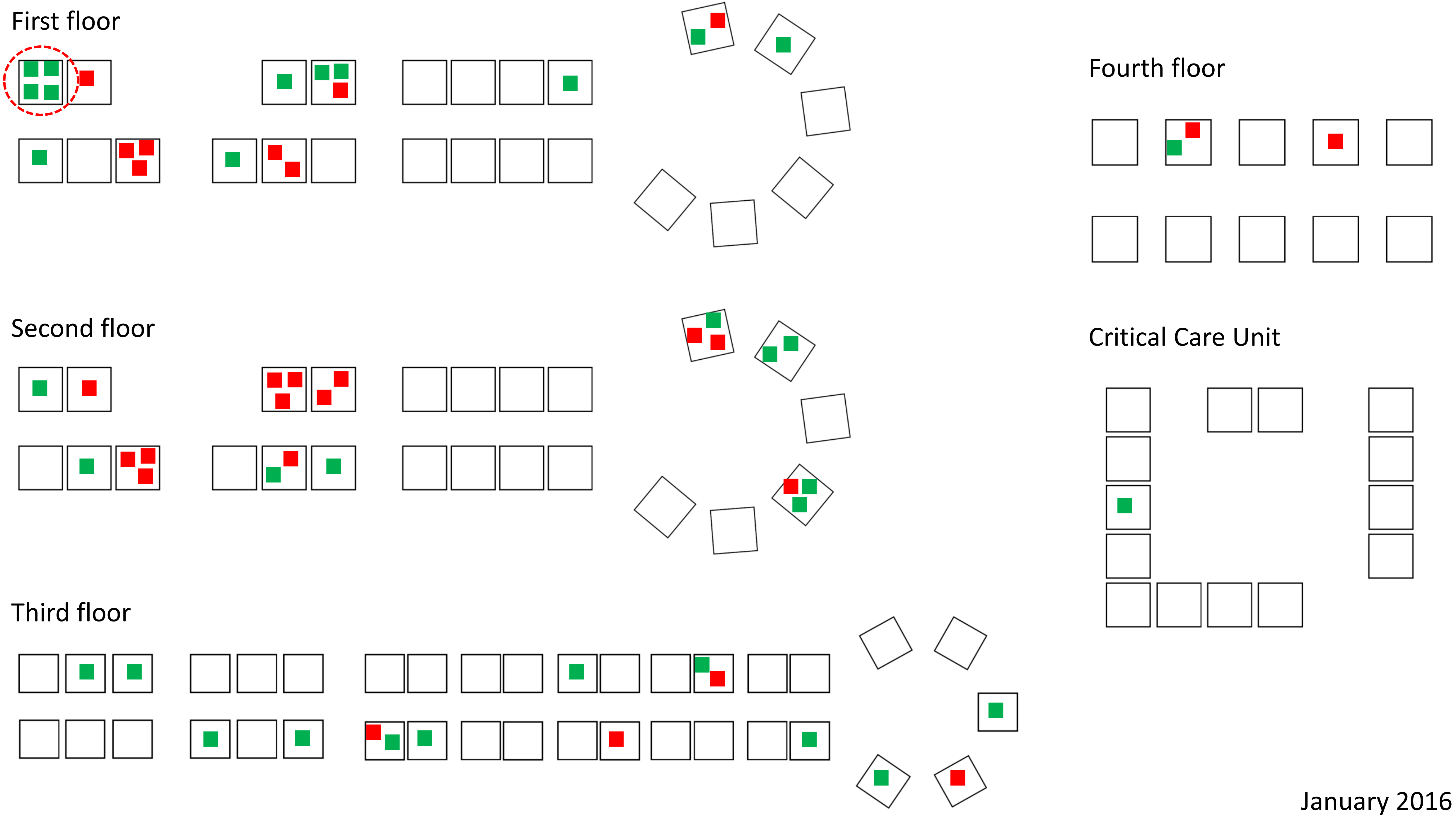
First floor
Fourth floor
Second floor
Critical Care Unit
Third floor
January 2016

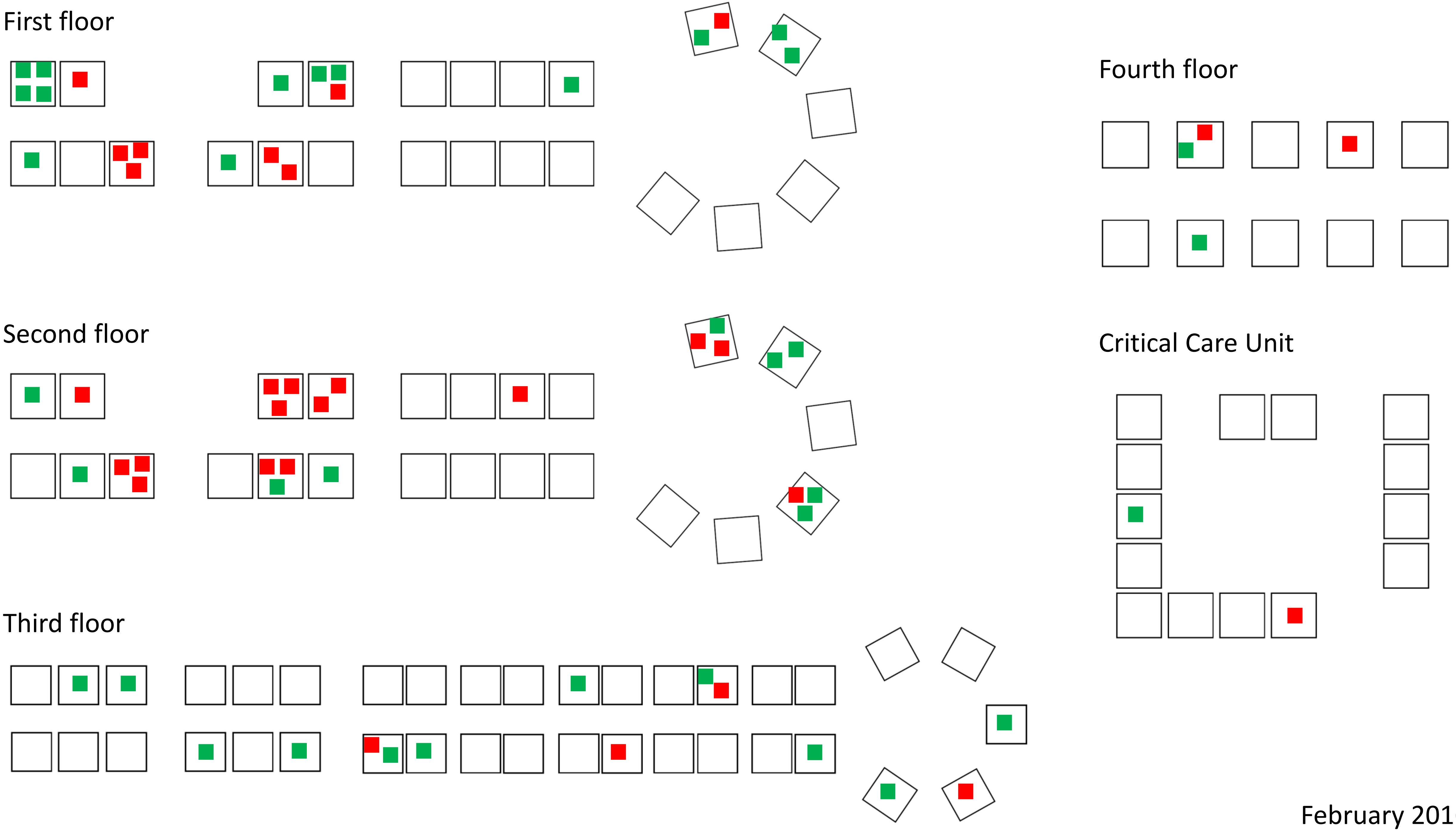

February 2016

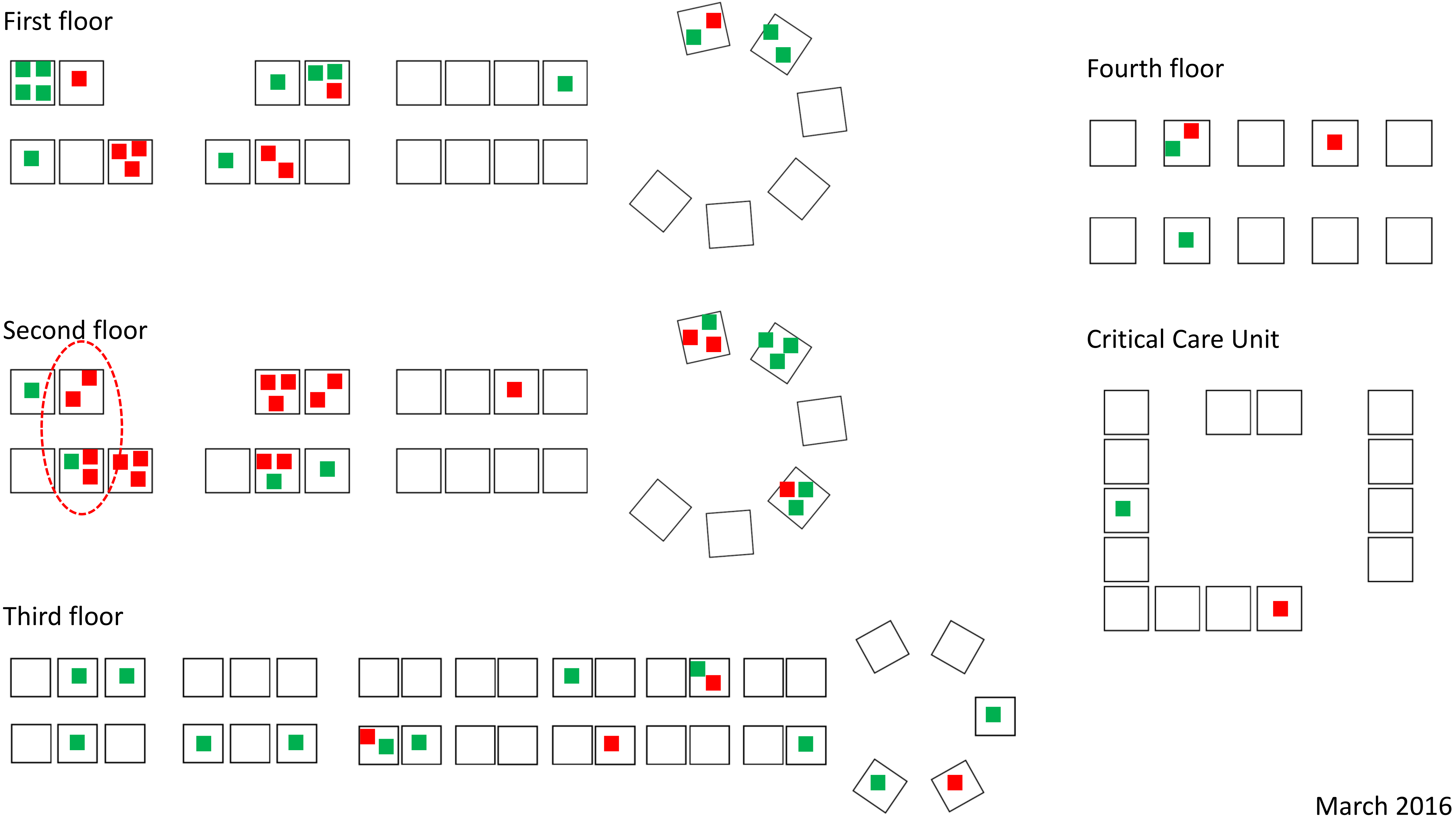
First floor
Second floor
Third floor
Fourth floor
Critical Care Unit
March 2016

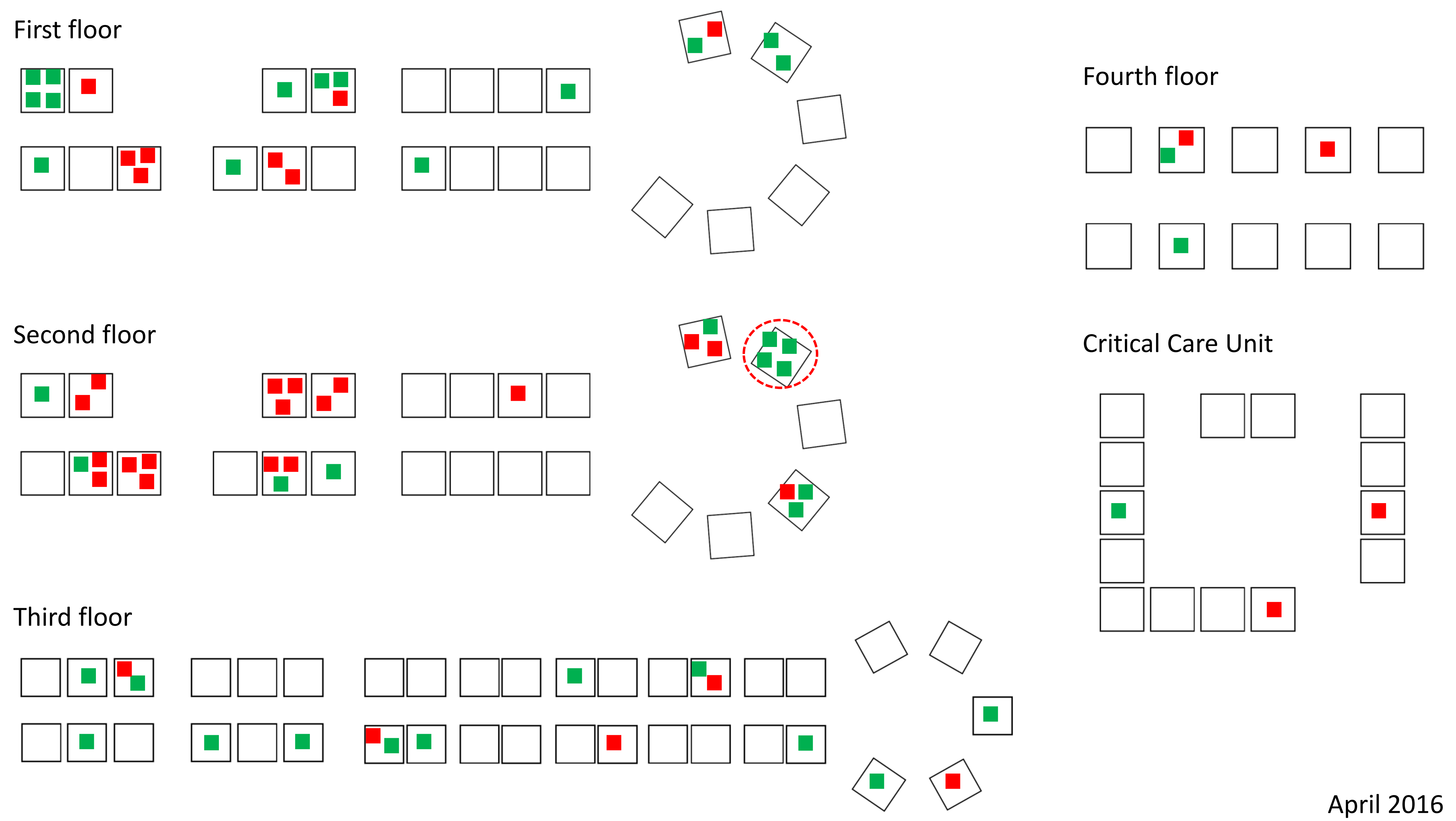
First floor
Second floor
Third floor
Fourth floor
Critical Care Unit
April 2016

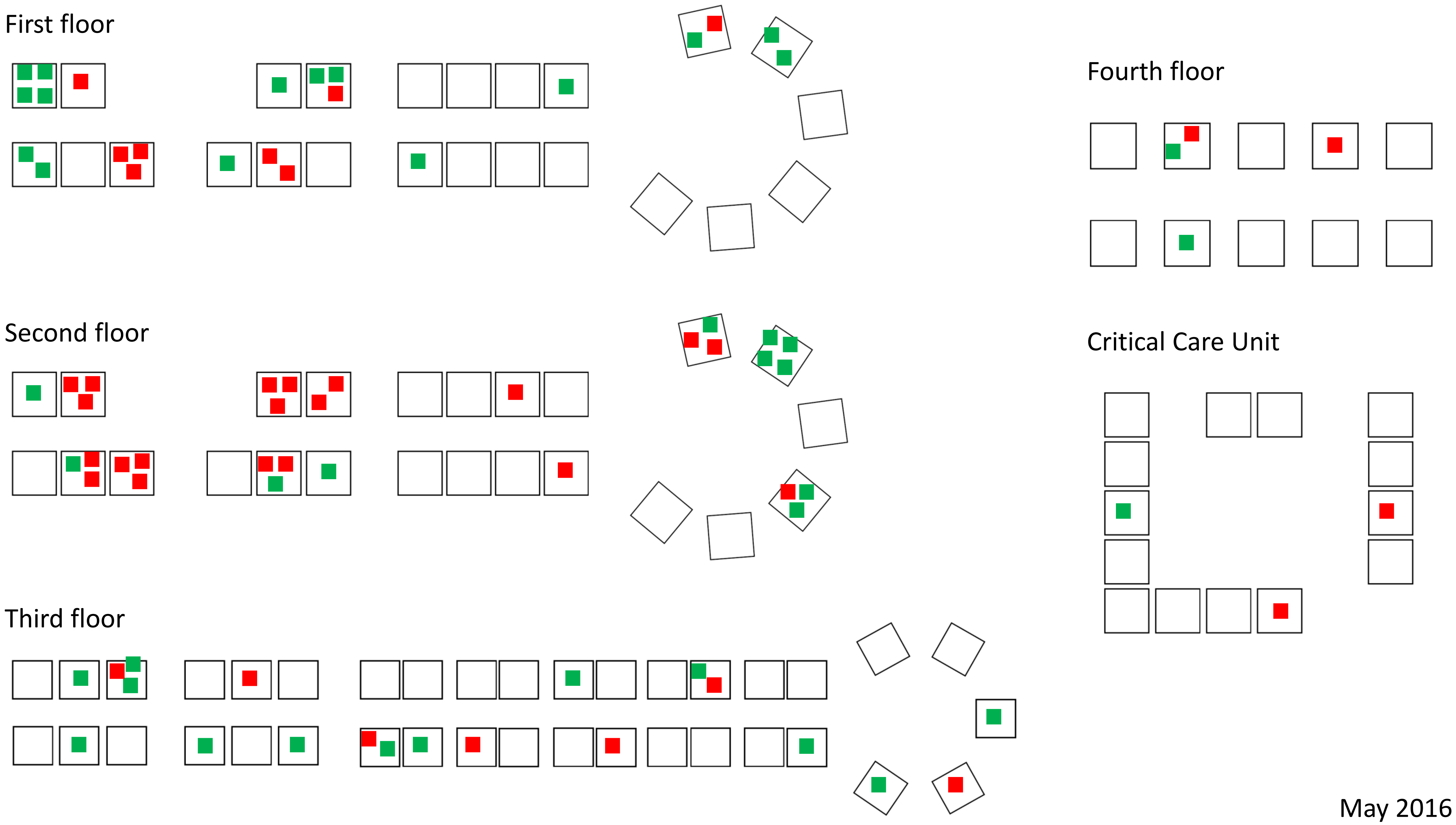
First floor
Fourth floor
Second floor
Critical Care Unit
Third floor
May 2016

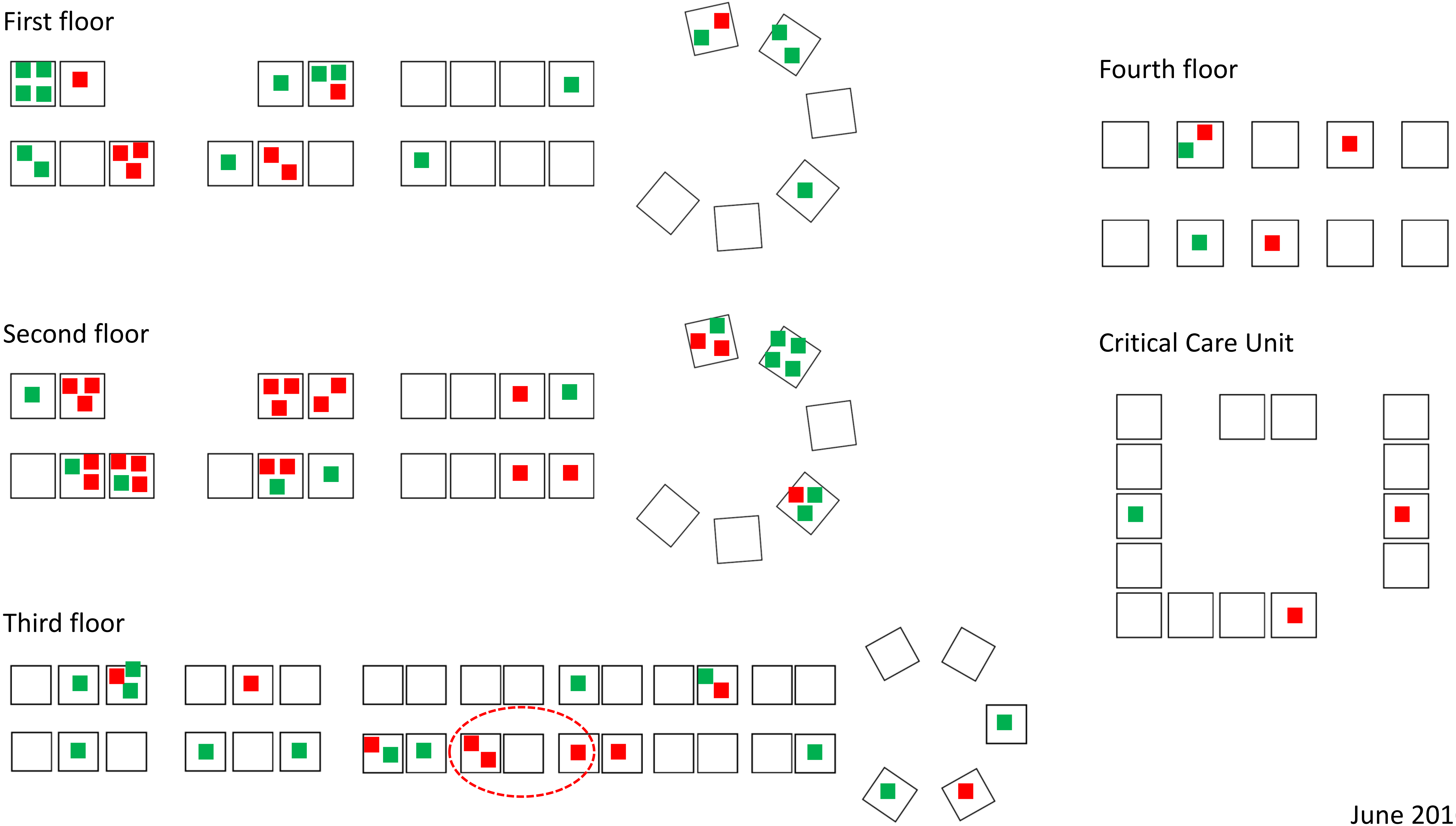
First floor
Second floor
Third floor
Fourth floor
Critical Care Unit

June 2016

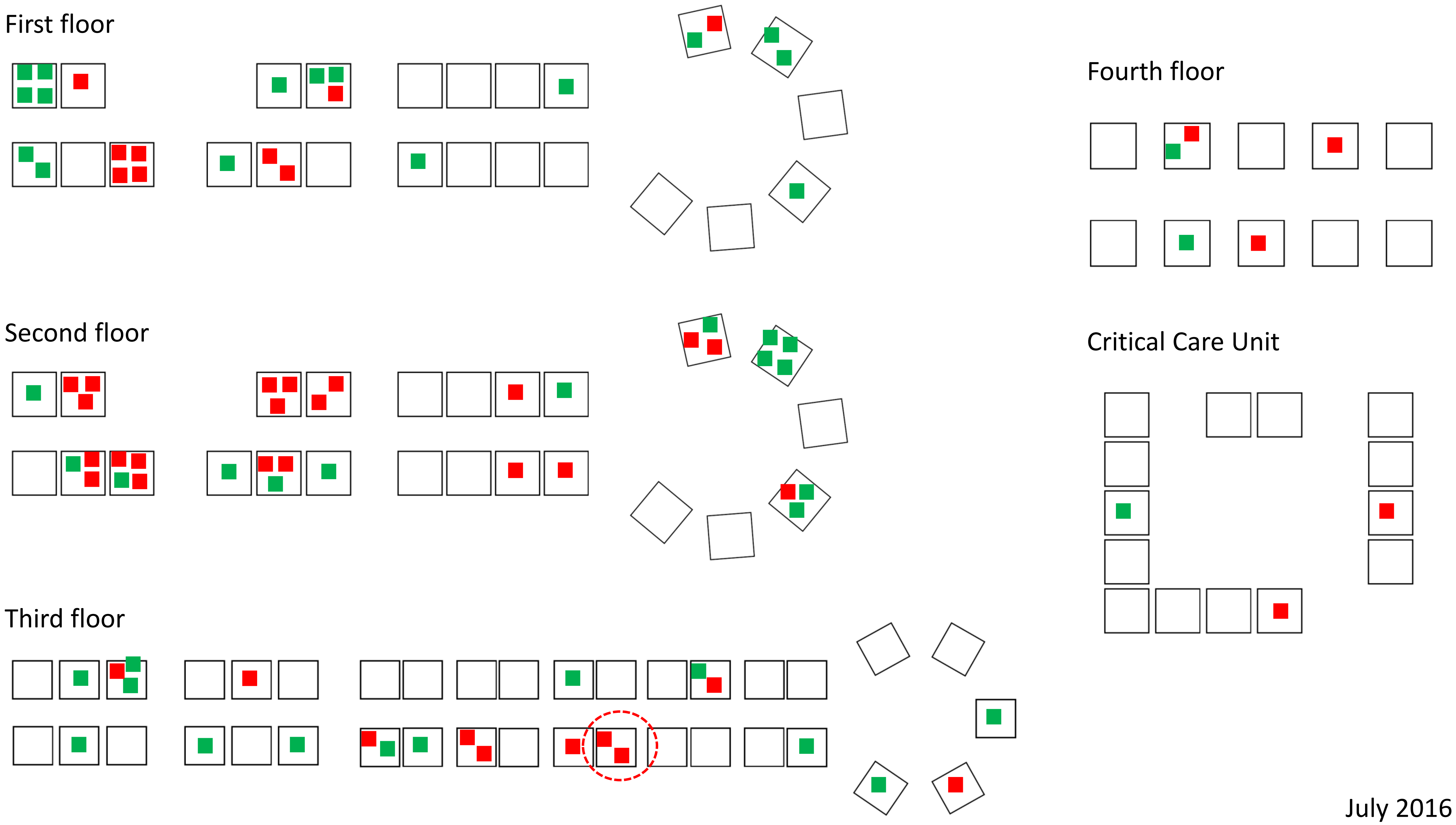

First floor
Second floor
Third floor
Fourth floor
Critical Care Unit
July 2016

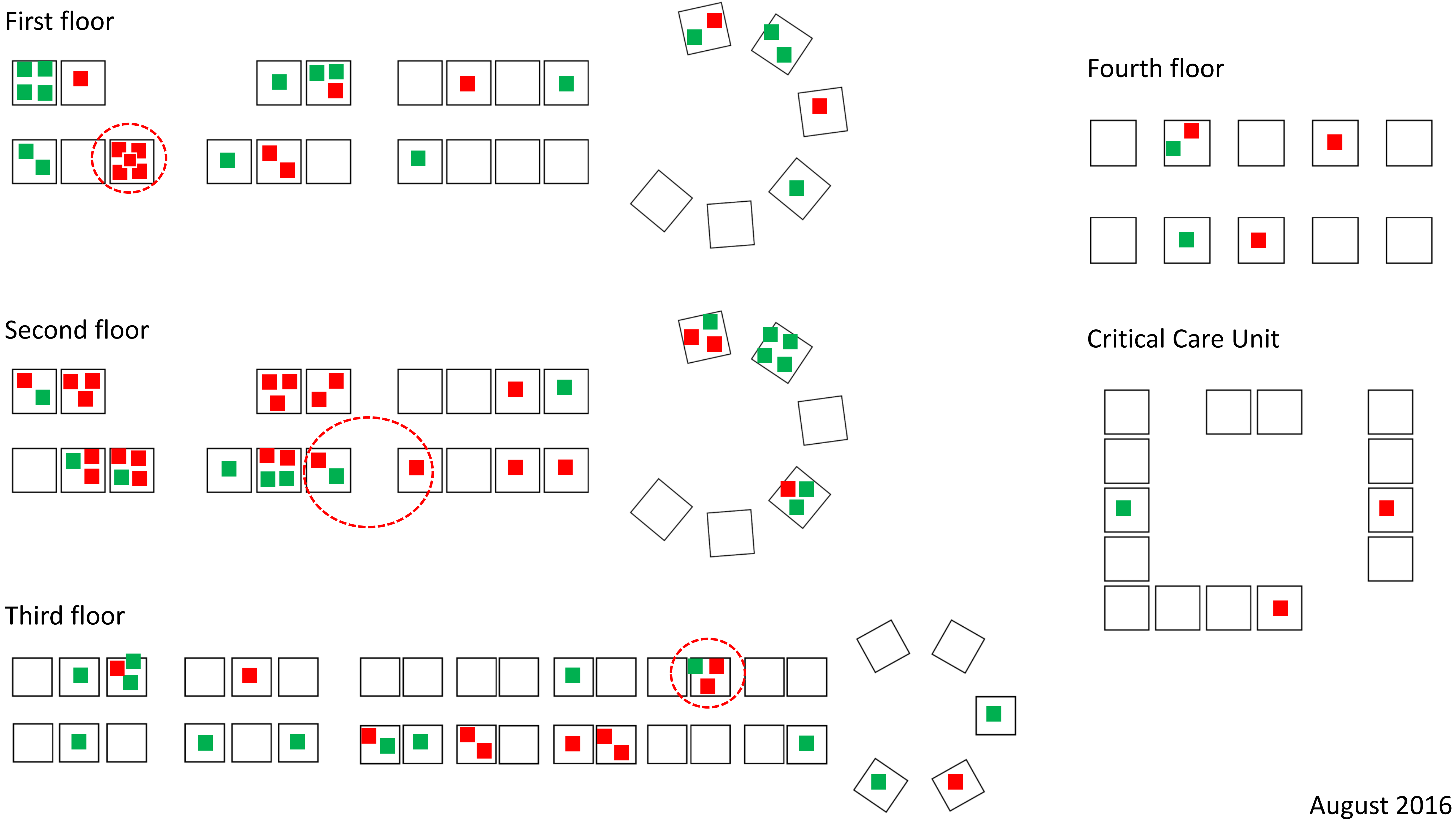
First floor
Fourth floor
Second floor
Critical Care Unit
Third floor
August 2016

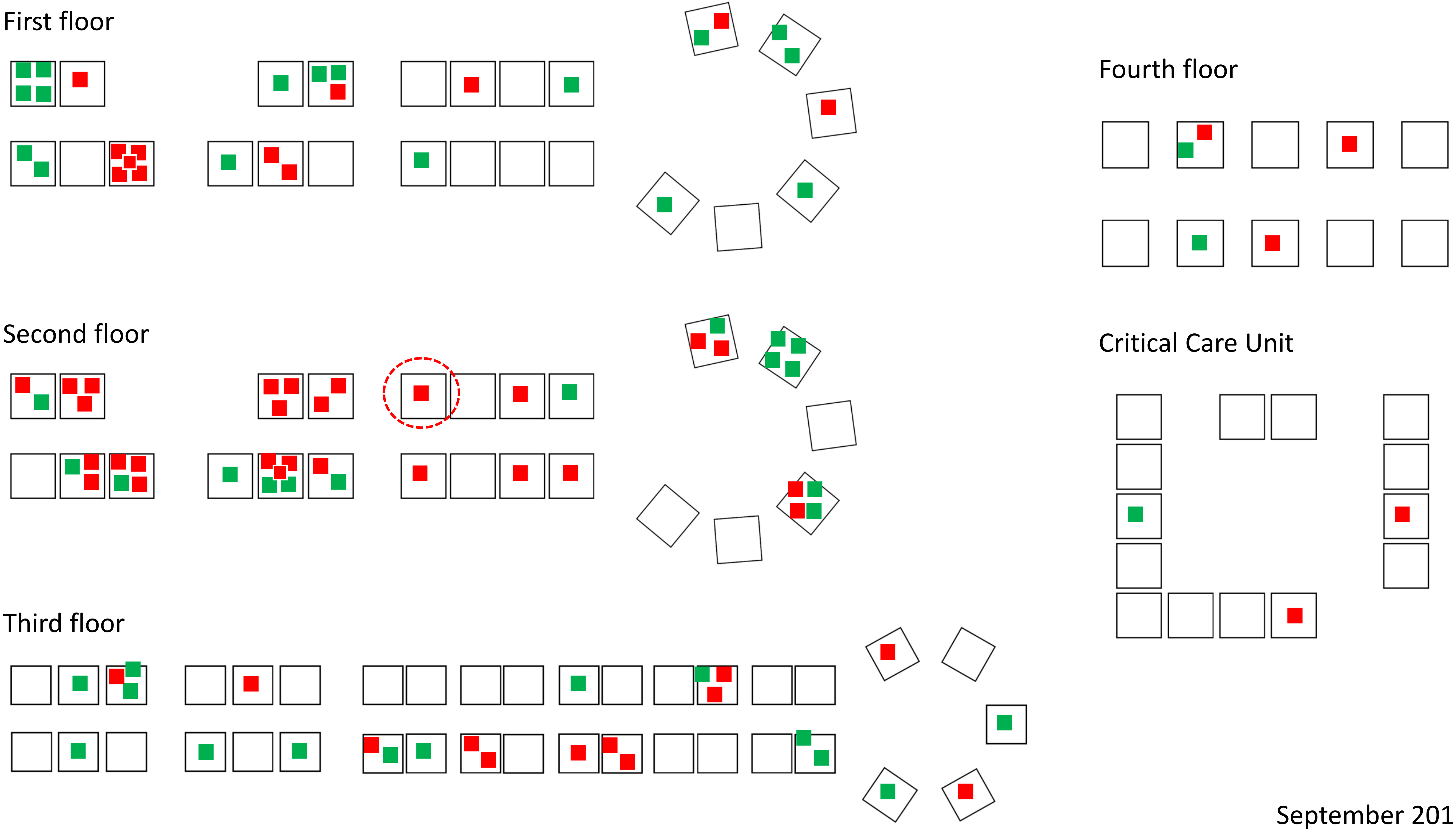

September 2016

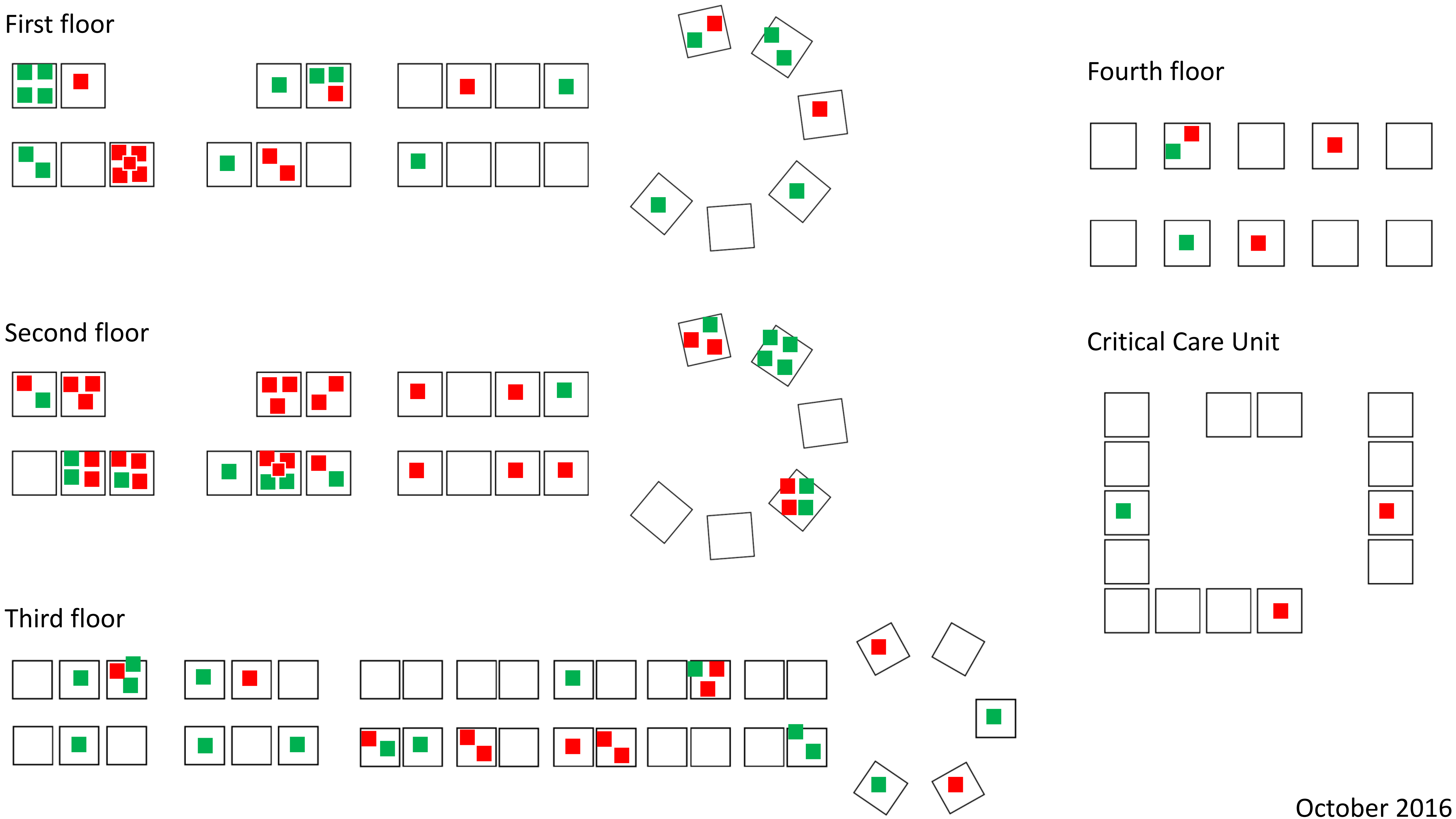
First floor
Second floor
Third floor
Fourth floor
Critical Care Unit
October 2016

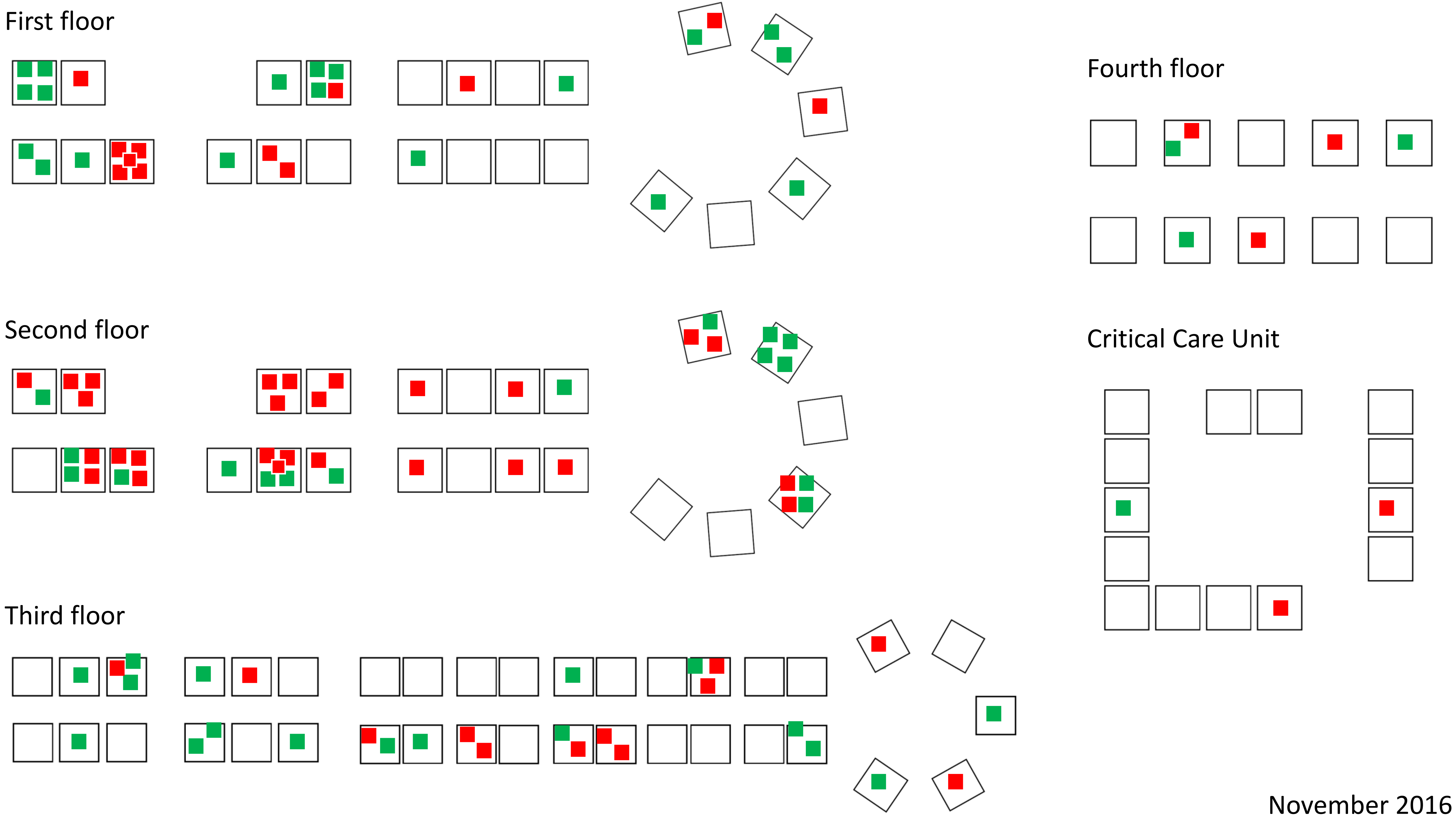
First floor
Second floor
Third floor
Fourth floor
Critical Care Unit
November 2016

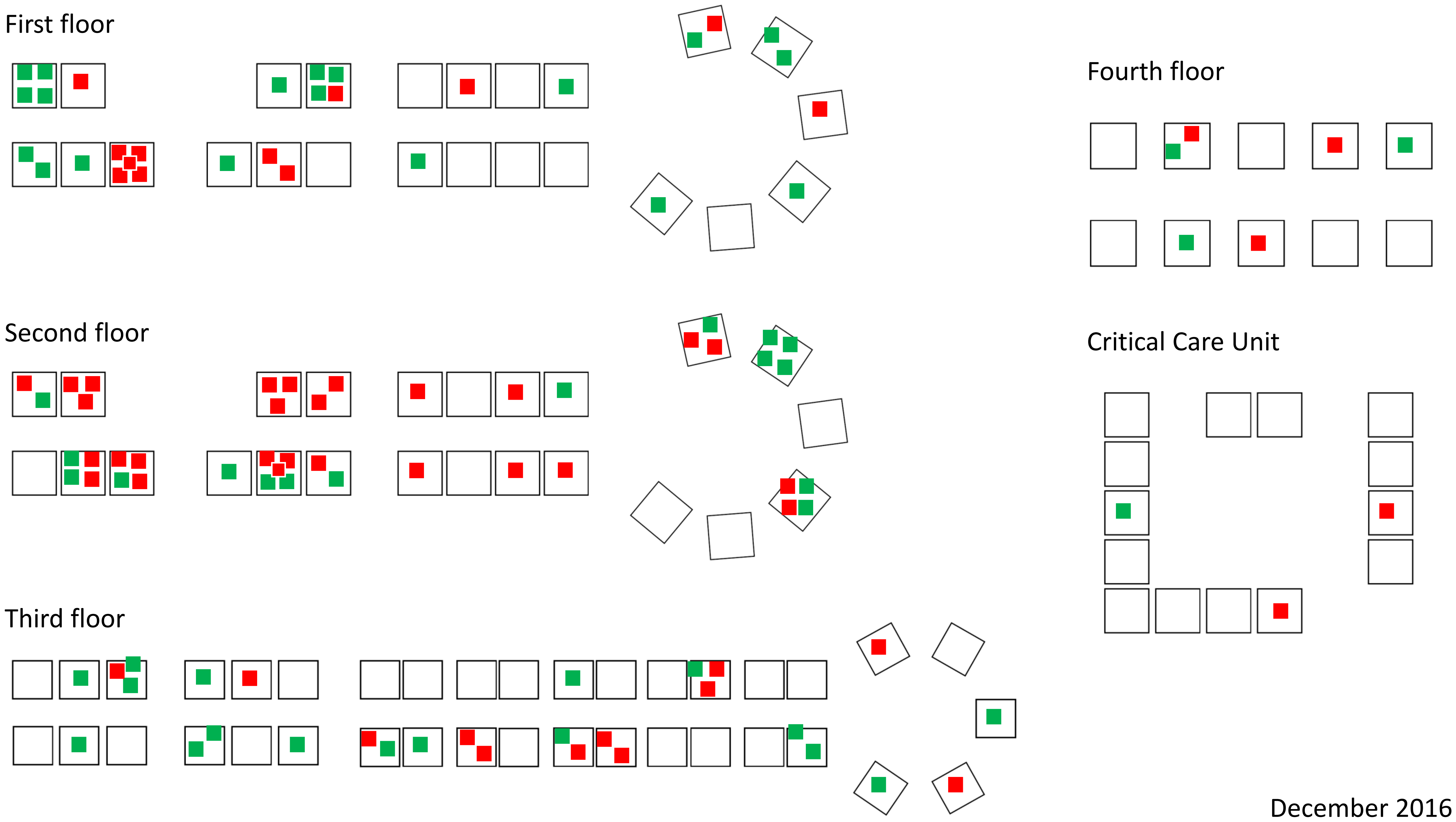
First floor
Second floor
Third floor
Fourth floor
Critical Care Unit
December 2016
