# Supplementary material for: Factors associated with an outbreak of hospital-onset, healthcare facility-associated Clostridium difficile infection (HO-HCFA CDI) in a Mexican tertiary care hospital: A case-control study
Source: PLoS One. 2018 May 29;13(5):e0198212. doi: 10.1371/journal.pone.0198212 (PMC5973614; doi:10.1371/journal.pone.0198212)
Supplement: S3 File — (PDF) [file pone.0198212.s003.pdf]

Purposeful search of interactions between antibiotics and PPI.

```
. use "/Users/ericochoahein/Desktop/stata database.dta"

. cc diarrhea quinol,by(ppi)

             ppi |       OR       [95% Conf. Interval]   M-H Weight
-----------------+-------------------------------------------------
               0 |     1.3625      .2101834   6.733116     1.518987
(exact)
               1 |   2.261029      1.033582   4.987054     4.792952
(exact)
-----------------+-------------------------------------------------
           Crude |   2.245791      1.138686   4.418238
(exact)
    M-H combined |   2.044796      1.079736    3.87242
-------------------------------------------------------------------
Test of homogeneity (M-H)      chi2(1) =     0.38  Pr>chi2 = 0.5359

                   Test that combined OR = 1:
                                Mantel-Haenszel chi2(1) =      4.88
                                                Pr>chi2 =    0.0272

. cc diarrhea cipro,by(ppi)

             ppi |       OR       [95% Conf. Interval]   M-H Weight
-----------------+-------------------------------------------------
               0 |   .6607143      .0131084   6.946825     1.063291
(exact)
               1 |   11.87333      2.557959   110.0206      .660793
(exact)
-----------------+-------------------------------------------------
           Crude |   4.945869      1.723975   16.03627
(exact)
    M-H combined |   4.958196      1.803404   13.63184
-------------------------------------------------------------------
Test of homogeneity (M-H)      chi2(1) =     4.44  Pr>chi2 = 0.0351

                   Test that combined OR = 1:
                                Mantel-Haenszel chi2(1) =     10.82
                                                Pr>chi2 =    0.0010

. cc diarrhea moxi,by(ppi)

             ppi |       OR       [95% Conf. Interval]   M-H Weight
-----------------+-------------------------------------------------
               0 |   2.714286      .0337809    214.881     .2658228
(exact)
               1 |   .3359173      .0346555   1.685869     3.409692
(exact)
-----------------+-------------------------------------------------
           Crude |    .571875      .0995344   2.276105
(exact)
    M-H combined |   .5079271      .1396106   1.847926
-------------------------------------------------------------------
Test of homogeneity (M-H)      chi2(1) =     1.65  Pr>chi2 = 0.1985
```

```
                   Test that combined OR = 1:
                                Mantel-Haenszel chi2(1) =      1.13
                                                Pr>chi2 =    0.2873

. cc diarrhea levo,by(ppi)

             ppi |       OR       [95% Conf. Interval]   M-H Weight
-----------------+-------------------------------------------------
               0 |   2.714286      .0337809    214.881     .2658228
(exact)
               1 |   1.960976      .4801612   8.369381     1.806167
(exact)
-----------------+-------------------------------------------------
           Crude |   2.333333      .6543785   8.575183
(exact)
    M-H combined |    2.05762      .6725257   6.295375
-------------------------------------------------------------------
Test of homogeneity (M-H)      chi2(1) =     0.04  Pr>chi2 = 0.8343

                   Test that combined OR = 1:
                                Mantel-Haenszel chi2(1) =      1.66
                                                Pr>chi2 =    0.1973

. cc diarrhea mero,by(ppi)

             ppi |       OR       [95% Conf. Interval]   M-H Weight
-----------------+-------------------------------------------------
               0 |   1.668981      .5131656   5.015426     2.734177
(exact)
               1 |   3.301587      1.746941   6.256765     5.828194
(exact)
-----------------+-------------------------------------------------
           Crude |   2.983409      1.763253   5.045414
(exact)
    M-H combined |   2.780256      1.677488   4.607974
-------------------------------------------------------------------
Test of homogeneity (M-H)      chi2(1) =     1.31  Pr>chi2 = 0.2526

                   Test that combined OR = 1:
                                Mantel-Haenszel chi2(1) =     16.03
                                                Pr>chi2 =    0.0001

. cc diarrhea meroimi,by(ppi)

             ppi |       OR       [95% Conf. Interval]   M-H Weight
-----------------+-------------------------------------------------
               0 |   1.636364      .6557448   3.927728     4.316456
(exact)
               1 |    2.67907      1.485492   4.834393     7.577093
(exact)
-----------------+-------------------------------------------------
           Crude |   2.457205      1.535833    3.92413
(exact)
    M-H combined |   2.300647      1.462187   3.619903
-------------------------------------------------------------------
Test of homogeneity (M-H)      chi2(1) =     0.97  Pr>chi2 = 0.3253
```

```
                   Test that combined OR = 1:
                                Mantel-Haenszel chi2(1) =     13.06
                                                Pr>chi2 =    0.0003

. cc diarrhea erta,by(ppi)

             ppi |       OR       [95% Conf. Interval]   M-H Weight
-----------------+-------------------------------------------------
               0 |   1.605735      .7057065   3.574171     5.297468
(exact)
               1 |   2.050493      1.069419   3.926246     7.154185
(exact)
-----------------+-------------------------------------------------
           Crude |   1.788634      1.094778   2.909751
(exact)
    M-H combined |   1.861274      1.165446   2.972543
-------------------------------------------------------------------
Test of homogeneity (M-H)      chi2(1) =     0.25  Pr>chi2 = 0.6170

                   Test that combined OR = 1:
                                Mantel-Haenszel chi2(1) =      6.81
                                                Pr>chi2 =    0.0090

. cc diarrhea carba,by(ppi)

             ppi |       OR       [95% Conf. Interval]   M-H Weight
-----------------+-------------------------------------------------
               0 |   2.083333      .9647007    4.53079     5.240506
(exact)
               1 |   2.401075      1.333549   4.347545     8.193833
(exact)
-----------------+-------------------------------------------------
           Crude |   2.337577       1.48199   3.698481
(exact)
    M-H combined |    2.27713      1.471658   3.523453
-------------------------------------------------------------------
Test of homogeneity (M-H)      chi2(1) =     0.10  Pr>chi2 = 0.7576

                   Test that combined OR = 1:
                                Mantel-Haenszel chi2(1) =     13.83
                                                Pr>chi2 =    0.0002

. cc diarrhea vanco,by(ppi)

             ppi |       OR       [95% Conf. Interval]   M-H Weight
-----------------+-------------------------------------------------
               0 |   2.135172      .9480461   4.741831     4.588608
(exact)
               1 |   1.703607      .9600389   3.026779     10.13656
(exact)
-----------------+-------------------------------------------------
           Crude |   1.974787       1.25611   3.102246
(exact)
    M-H combined |    1.83809      1.189113   2.841257
-------------------------------------------------------------------
Test of homogeneity (M-H)      chi2(1) =     0.23  Pr>chi2 = 0.6279
```

```
                   Test that combined OR = 1:
                                Mantel-Haenszel chi2(1) =      7.58
                                                Pr>chi2 =    0.0059

. cc diarrhea ptz,by(ppi)

             ppi |       OR       [95% Conf. Interval]   M-H Weight
-----------------+-------------------------------------------------
               0 |   2.142857      .9050751   4.956107     4.075949
(exact)
               1 |   1.545617      .8513172   2.797575      9.85022
(exact)
-----------------+-------------------------------------------------
           Crude |   1.815909      1.127745   2.913813
(exact)
    M-H combined |   1.720419      1.093902   2.705765
-------------------------------------------------------------------
Test of homogeneity (M-H)      chi2(1) =     0.45  Pr>chi2 = 0.5024

                   Test that combined OR = 1:
                                Mantel-Haenszel chi2(1) =      5.58
                                                Pr>chi2 =    0.0182

. cc diarrhea ceftria,by(ppi)

             ppi |       OR       [95% Conf. Interval]   M-H Weight
-----------------+-------------------------------------------------
               0 |   .9348172      .2799157   2.732462     3.981013
(exact)
               1 |   1.681159       .822361   3.418451     6.687225
(exact)
-----------------+-------------------------------------------------
           Crude |   1.454369      .8146363   2.567542
(exact)
    M-H combined |   1.402651      .8143603   2.415919
-------------------------------------------------------------------
Test of homogeneity (M-H)      chi2(1) =     0.92  Pr>chi2 = 0.3381

                   Test that combined OR = 1:
                                Mantel-Haenszel chi2(1) =      1.47
                                                Pr>chi2 =    0.2247

. cc diarrhea cefta,by(ppi)

             ppi |       OR       [95% Conf. Interval]   M-H Weight
-----------------+-------------------------------------------------
               0 |   2.756098      .1925157   38.87363     .5189873
(exact)
               1 |          .      1.254594          .            0
(exact)
-----------------+-------------------------------------------------
           Crude |          5      .8014336   52.95519
(exact)
    M-H combined |   6.295691      1.098197   36.09163
-------------------------------------------------------------------
Test of homogeneity (Tarone)   chi2(1) =     1.46  Pr>chi2 = 0.2275
```

```
                   Test that combined OR = 1:
                                Mantel-Haenszel chi2(1) =      5.09
                                                Pr>chi2 =    0.0240

. cc diarrhea cefalosp3rd,by(ppi)

             ppi |       OR       [95% Conf. Interval]   M-H Weight
-----------------+-------------------------------------------------
               0 |   1.231746      .4234538   3.304982     3.987342
(exact)
               1 |    2.00138      .9979315   4.010815      6.38326
(exact)
-----------------+-------------------------------------------------
           Crude |   1.749378      1.005333   3.024267
(exact)
    M-H combined |   1.705467      1.011369   2.875921
-------------------------------------------------------------------
Test of homogeneity (M-H)      chi2(1) =     0.72  Pr>chi2 = 0.3963

                   Test that combined OR = 1:
                                Mantel-Haenszel chi2(1) =      4.01
                                                Pr>chi2 =    0.0453

. cc diarrhea clinda,by(ppi)

             ppi |       OR       [95% Conf. Interval]   M-H Weight
-----------------+-------------------------------------------------
               0 |          0             0    5.18673     .5443038
(exact)
               1 |   1.614458      .3590788   7.226567     1.828194
(exact)
-----------------+-------------------------------------------------
           Crude |   1.400227      .3429227   5.237554
(exact)
    M-H combined |   1.244065      .3777085     4.0976
-------------------------------------------------------------------
Test of homogeneity (Tarone)   chi2(1) =     1.10  Pr>chi2 = 0.2935

                   Test that combined OR = 1:
                                Mantel-Haenszel chi2(1) =      0.13
                                                Pr>chi2 =    0.7232

.
```

Purposeful search of interactions between different variables. Includes new variables comprising interaction terms between antibiotics.

```
. use "/Users/ericochoahein/Desktop/stata database.dta"

. cc diarrhea ciprointraabd

                 | ciprointraabd          |           Proportion
                 |   Exposed   Unexposed  |      Total     Exposed
-----------------+------------------------+------------------------
           Cases |         1         146  |        147       0.0068
        Controls |         0         272  |        272       0.0000
-----------------+------------------------+------------------------
           Total |         1         418  |        419       0.0024
                 |                        |
                 |      Point estimate    |    [95% Conf. Interval]
                 |------------------------+------------------------
      Odds ratio |                .       |          0           .
(Cornfie
> ld)
 Attr. frac. ex. |                1       |          .           .
(Cornfie
> ld)
 Attr. frac. pop |         .0068027       |
                 +-------------------------------------------------
                               chi2(1) =     1.85  Pr>chi2 = 0.1732

 Note: Exact confidence levels not possible with zero count cells.

. cc diarrhea cipro_feb_neutr

                 | cipro_feb_neutr        |           Proportion
                 |   Exposed   Unexposed  |      Total     Exposed
-----------------+------------------------+------------------------
           Cases |         3         144  |        147       0.0204
        Controls |         0         272  |        272       0.0000
-----------------+------------------------+------------------------
           Total |         3         416  |        419       0.0072
                 |                        |
                 |      Point estimate    |    [95% Conf. Interval]
                 |------------------------+------------------------
      Odds ratio |                .       |    1.460496            .
(Cornfield)
 Attr. frac. ex. |                1       |    .3153012            .
(Cornfield)
 Attr. frac. pop |         .0204082       |
                 +-------------------------------------------------
                               chi2(1) =     5.59  Pr>chi2 = 0.0181

 Note: Exact confidence levels not possible with zero count cells.

. cc diarrhea cipro,by(feb_neutr)

       feb_neutr |       OR       [95% Conf. Interval]   M-H Weight
-----------------+-------------------------------------------------
```

```
                0 |     4.7125      1.617463   15.42872          1.8
(exact)
                1 |          .      .2783276          .            0
(exact)
-----------------+-------------------------------------------------
            Crude |   5.414758      1.947733    17.2212
(exact)
     M-H combined |   5.151096      1.918599   13.82978
-------------------------------------------------------------------
Test of homogeneity (Tarone)   chi2(1) =     0.29  Pr>chi2 = 0.5897

                   Test that combined OR = 1:
                                Mantel-Haenszel chi2(1) =     12.29
                                                Pr>chi2 =    0.0005

. cc diarrhea intraabd_feb_neutr

                 | intraabd_feb_neutr     |             Proportion
                 |   Exposed   Unexposed  |      Total     Exposed
-----------------+------------------------+------------------------
           Cases |         0         148  |        148       0.0000
        Controls |         0         297  |        297       0.0000
-----------------+------------------------+------------------------
           Total |         0         445  |        445       0.0000
                 |                        |
                 |      Point estimate    |    [95% Conf. Interval]
                 |------------------------+------------------------
      Odds ratio |                 .      |          .           .
(Cornfield)
 Attr. frac. ex. |                 .      |          .           .
(Cornfield)
 Attr. frac. pop |                 .      |
                 +-------------------------------------------------
                               chi2(1) =        .  Pr>chi2 =      .

 Note: Exact confidence levels not possible with zero count cells.

. cc diarrhea intraabd_ppi
                                                         Proportion
                 |   Exposed   Unexposed  |      Total     Exposed
-----------------+------------------------+------------------------
           Cases |         5         126  |        131       0.0382
        Controls |         4         265  |        269       0.0149
-----------------+------------------------+------------------------
           Total |         9         391  |        400       0.0225
                 |                        |
                 |      Point estimate    |    [95% Conf. Interval]
                 |------------------------+------------------------
      Odds ratio |         2.628968       |    .5539917    13.44804
(exact)
 Attr. frac. ex. |         .6196226       |   -.8050813    .9256397
(exact)
 Attr. frac. pop |         .0236497       |
                 +-------------------------------------------------
                               chi2(1) =     2.17  Pr>chi2 = 0.1403

. cc diarrhea intraabd_surg
```

```
                                                         Proportion
                 |   Exposed   Unexposed  |      Total     Exposed
-----------------+------------------------+------------------------
           Cases |         8         135  |        143       0.0559
        Controls |         4         285  |        289       0.0138
-----------------+------------------------+------------------------
           Total |        12         420  |        432       0.0278
                 |                        |
                 |      Point estimate    |    [95% Conf. Interval]
                 |------------------------+------------------------
      Odds ratio |         4.222222       |    1.104079    19.42873
(exact)
 Attr. frac. ex. |         .7631579       |    .0942679    .9485298
(exact)
 Attr. frac. pop |         .0426941       |
                 +-------------------------------------------------
                               chi2(1) =     6.28  Pr>chi2 = 0.0122

. cc diarrhea intraabdsepsis,by(surgery)

         surgery |       OR       [95% Conf. Interval]   M-H Weight
-----------------+-------------------------------------------------
               0 |   1.337662      .2796655   5.414059     2.019672
(exact)
               1 |   4.136364       1.64258   10.68635     2.598425
(exact)
-----------------+-------------------------------------------------
           Crude |   3.189736      1.599003   6.444027
(exact)
    M-H combined |   2.912384      1.478102   5.738425
-------------------------------------------------------------------
Test of homogeneity (M-H)      chi2(1) =     2.14  Pr>chi2 = 0.1438

                   Test that combined OR = 1:
                                Mantel-Haenszel chi2(1) =      9.70
                                                Pr>chi2 =    0.0018

. cc diarrhea meroimicipro
                                                         Proportion
                 |   Exposed   Unexposed  |      Total     Exposed
-----------------+------------------------+------------------------
           Cases |        10         137  |        147       0.0680
        Controls |         5         270  |        275       0.0182
-----------------+------------------------+------------------------
           Total |        15         407  |        422       0.0355
                 |                        |
                 |      Point estimate    |    [95% Conf. Interval]
                 |------------------------+------------------------
      Odds ratio |         3.941606       |    1.195084    14.94298
(exact)
 Attr. frac. ex. |         .7462963       |    .1632387    .9330789
(exact)
 Attr. frac. pop |         .0507685       |
                 +-------------------------------------------------
                               chi2(1) =     6.94  Pr>chi2 = 0.0084

. cc diarrhea meroimi,by(cipro)
```

```
           cipro |       OR       [95% Conf. Interval]   M-H Weight
-----------------+-------------------------------------------------
               0 |   2.164251      1.356844   3.442482      12.9375
(exact)
               1 |   .3333333      .0060548   4.375056     1.363636
(exact)
-----------------+-------------------------------------------------
           Crude |   2.213213      1.420834   3.441456
(exact)
    M-H combined |    1.98967      1.295438   3.055944
-------------------------------------------------------------------
Test of homogeneity (M-H)      chi2(1) =     2.32  Pr>chi2 = 0.1281

                   Test that combined OR = 1:
                                Mantel-Haenszel chi2(1) =     10.03
                                                Pr>chi2 =    0.0015

. cc diarrhea meroimi_feb_neutr
                                                         Proportion
                 |   Exposed   Unexposed  |      Total     Exposed
-----------------+------------------------+------------------------
           Cases |        12         135  |        147       0.0816
        Controls |         4         268  |        272       0.0147
-----------------+------------------------+------------------------
           Total |        16         403  |        419       0.0382
                 |                        |
                 |      Point estimate    |    [95% Conf. Interval]
                 |------------------------+------------------------
      Odds ratio |         5.955556       |    1.754524    25.70165
(exact)
 Attr. frac. ex. |         .8320896       |    .4300449     .961092
(exact)
 Attr. frac. pop |         .0679257       |
                 +-------------------------------------------------
                               chi2(1) =    11.64  Pr>chi2 = 0.0006

. cc diarrhea meroimi,by(feb_neutr)

       feb_neutr |       OR       [95% Conf. Interval]   M-H Weight
-----------------+-------------------------------------------------
               0 |   1.923689      1.206585   3.057138       13.825
(exact)
               1 |        1.5      .0203848   36.15853     .4210526
(exact)
-----------------+-------------------------------------------------
           Crude |    2.18018       1.39912   3.391425
(exact)
    M-H combined |   1.911167      1.237652   2.951198
-------------------------------------------------------------------
Test of homogeneity (M-H)      chi2(1) =     0.03  Pr>chi2 = 0.8562

                   Test that combined OR = 1:
                                Mantel-Haenszel chi2(1) =      8.62
                                                Pr>chi2 =    0.0033

. cc diarrhea meroimiintraabd
```

```
                                                         Proportion
                 |   Exposed   Unexposed  |      Total     Exposed
-----------------+------------------------+------------------------
           Cases |         7         140  |        147       0.0476
        Controls |         6         266  |        272       0.0221
-----------------+------------------------+------------------------
           Total |        13         406  |        419       0.0310
                 |                        |
                 |      Point estimate    |    [95% Conf. Interval]
                 |------------------------+------------------------
      Odds ratio |         2.216667       |    .6232977    8.130992
(exact)
 Attr. frac. ex. |         .5488722       |   -.6043697    .8770138
(exact)
 Attr. frac. pop |         .0261368       |
                 +-------------------------------------------------
                              chi2(1) =     2.07  Pr>chi2 = 0.1499

. cc diarrhea cipro_feb_neutr_meroimi

                 | cipro_feb_neutr_meroimi|           Proportion
                 |   Exposed   Unexposed  |      Total     Exposed
-----------------+------------------------+------------------------
           Cases |         2         145  |        147       0.0136
        Controls |         0         272  |        272       0.0000
-----------------+------------------------+------------------------
           Total |         2         417  |        419       0.0048
                 |                        |
                 |      Point estimate    |    [95% Conf. Interval]
                 |------------------------+------------------------
      Odds ratio |                .       |    .9684111           .
(Cornfield)
 Attr. frac. ex. |                1       |   -.0326193           .
(Cornfield)
 Attr. frac. pop |         .0136054       |
                 +-------------------------------------------------
                              chi2(1) =     3.72  Pr>chi2 = 0.0538

 Note: Exact confidence levels not possible with zero count cells.

. cc diarrhea meroimi,by(cipro_feb_neutr)

    cipro_feb_~r |       OR       [95% Conf. Interval]   M-H Weight
-----------------+-------------------------------------------------
               0 |   2.140541      1.369205   3.339955     14.23077
(exact)
               1 |          .             0          .            0
(exact)
-----------------+-------------------------------------------------
           Crude |    2.18018       1.39912   3.391425
(exact)
    M-H combined |   2.140541      1.401519   3.269249
-------------------------------------------------------------------
Test of homogeneity (Tarone)   chi2(1) =     0.00  Pr>chi2 = 1.0000

                   Test that combined OR = 1:
                                Mantel-Haenszel chi2(1) =     12.59
```

```
                                    Pr>chi2 =    0.0004

. cc diarrhea cefaquinol
                                                          Proportion
                 |   Exposed   Unexposed  |      Total     Exposed
-----------------+------------------------+------------------------
           Cases |         7         140  |        147       0.0476
        Controls |         2         273  |        275       0.0073
-----------------+------------------------+------------------------
           Total |         9         413  |        422       0.0213
                 |                        |
                 |      Point estimate    |    [95% Conf. Interval]
                 |------------------------+------------------------
      Odds ratio |         6.825          |    1.271202     67.8561
(exact)
 Attr. frac. ex. |      .8534799          |    .2133428    .9852629
(exact)
 Attr. frac. pop |      .0406419          |
                 +-------------------------------------------------
                               chi2(1) =     7.47  Pr>chi2 = 0.0063

. cc diarrhea cefalosp,by(quinol)

          quinol |       OR       [95% Conf. Interval]   M-H Weight
-----------------+-------------------------------------------------
               0 |   1.728165      .9884198   2.995433     10.34759
(exact)
               1 |   4.083333      .6446354   43.90702          .75
(exact)
-----------------+-------------------------------------------------
           Crude |   1.845679      1.099608   3.084141
(exact)
    M-H combined |   1.887333      1.153591   3.087772
-------------------------------------------------------------------
Test of homogeneity (M-H)      chi2(1) =     0.91  Pr>chi2 = 0.3405

                   Test that combined OR = 1:
                                Mantel-Haenszel chi2(1) =      6.43
                                                Pr>chi2 =    0.0112

. cc diarrhea cefaptz
                                                          Proportion
                 |   Exposed   Unexposed  |      Total     Exposed
-----------------+------------------------+------------------------
           Cases |        14         133  |        147       0.0952
        Controls |         7         268  |        275       0.0255
-----------------+------------------------+------------------------
           Total |        21         401  |        422       0.0498
                 |                        |
                 |      Point estimate    |    [95% Conf. Interval]
                 |------------------------+------------------------
      Odds ratio |      4.030075          |    1.475544    12.04781
(exact)
 Attr. frac. ex. |      .7518657          |    .3222838    .9169973
(exact)
 Attr. frac. pop |      .0716063          |
                 +-------------------------------------------------
```

```
                               chi2(1) =     9.87  Pr>chi2 = 0.0017

. cc diarrhea cefalosp,by(ptz)

             ptz |       OR       [95% Conf. Interval]   M-H Weight
-----------------+-------------------------------------------------
               0 |   1.654705      .8821577   3.062461     8.501695
(exact)
               1 |   3.047619      1.037433   9.624495     2.314961
(exact)
-----------------+-------------------------------------------------
           Crude |   1.845679      1.099608   3.084141
(exact)
    M-H combined |   1.952814      1.190973    3.20199
-------------------------------------------------------------------
Test of homogeneity (M-H)      chi2(1) =     1.09  Pr>chi2 = 0.2955

                   Test that combined OR = 1:
                                Mantel-Haenszel chi2(1) =      7.10
                                                Pr>chi2 =    0.0077

. cc diarrhea cefavanco
                                                         Proportion
                 |   Exposed   Unexposed  |      Total     Exposed
-----------------+------------------------+------------------------
           Cases |        23         124  |        147       0.1565
        Controls |        18         257  |        275       0.0655
-----------------+------------------------+------------------------
           Total |        41         381  |        422       0.0972
                 |                        |
                 |      Point estimate    |    [95% Conf. Interval]
                 |------------------------+------------------------
      Odds ratio |         2.648297       |    1.310843    5.403889
(exact)
 Attr. frac. ex. |         .6223989       |    .2371321    .8149481
(exact)
 Attr. frac. pop |         .0973821       |
                 +-------------------------------------------------
                               chi2(1) =     9.05  Pr>chi2 = 0.0026

. cc diarrhea cefalosp,by(vanco)

           vanco |       OR       [95% Conf. Interval]   M-H Weight
-----------------+-------------------------------------------------
               0 |   1.646091      .7644462   3.450017     5.902834
(exact)
               1 |   1.893004      .8804085   4.093233     5.554286
(exact)
-----------------+-------------------------------------------------
           Crude |   1.845679      1.099608   3.084141
(exact)
    M-H combined |   1.765792      1.077859   2.892791
-------------------------------------------------------------------
Test of homogeneity (M-H)      chi2(1) =     0.08  Pr>chi2 = 0.7818

                   Test that combined OR = 1:
                                Mantel-Haenszel chi2(1) =      5.12
```

```
                                                 Pr>chi2 =    0.0236

. cc diarrhea cefaclinda
                                                         Proportion
                 |   Exposed   Unexposed  |      Total     Exposed
-----------------+------------------------+------------------------
           Cases |         2         145  |        147       0.0136
        Controls |         2         273  |        275       0.0073
-----------------+------------------------+------------------------
           Total |         4         418  |        422       0.0095
                 |                        |
                 |      Point estimate    |    [95% Conf. Interval]
                 |------------------------+------------------------
      Odds ratio |         1.882759       |    .1349711     26.1786
(exact)
 Attr. frac. ex. |         .4688645       |   -6.408993    .9618009
(exact)
 Attr. frac. pop |         .0063791       |
                 +-------------------------------------------------
                               chi2(1) =     0.41  Pr>chi2 = 0.5224

. cc diarrhea ptzquinol
                                                         Proportion
                 |   Exposed   Unexposed  |      Total     Exposed
-----------------+------------------------+------------------------
           Cases |        11         136  |        147       0.0748
        Controls |        11         264  |        275       0.0400
-----------------+------------------------+------------------------
           Total |        22         400  |        422       0.0521
                 |                        |
                 |      Point estimate    |    [95% Conf. Interval]
                 |------------------------+------------------------
      Odds ratio |         1.941176       |    .7411584    5.069244
(exact)
 Attr. frac. ex. |         .4848485       |   -.3492393    .8027319
(exact)
 Attr. frac. pop |         .0362812       |
                 +-------------------------------------------------
                               chi2(1) =     2.35  Pr>chi2 = 0.1251

. cc diarrhea vancoquinol
                                                         Proportion
                 |   Exposed   Unexposed  |      Total     Exposed
-----------------+------------------------+------------------------
           Cases |        17         130  |        147       0.1156
        Controls |        15         260  |        275       0.0545
-----------------+------------------------+------------------------
           Total |        32         390  |        422       0.0758
                 |                        |
                 |      Point estimate    |    [95% Conf. Interval]
                 |------------------------+------------------------
      Odds ratio |         2.266667       |    1.026355    5.033862
(exact)
 Attr. frac. ex. |         .5588235       |    .0256783    .8013454
(exact)
 Attr. frac. pop |         .0646259       |
                 +-------------------------------------------------
```

```
                               chi2(1) =     5.10  Pr>chi2 = 0.0239

. cc diarrhea vanco,by(quinol)

          quinol |       OR       [95% Conf. Interval]   M-H Weight
-----------------+-------------------------------------------------
               0 |   1.970462      1.235753    3.13739     13.75936
(exact)
               1 |   1.133333      .2882673   4.450787          2.5
(exact)
-----------------+-------------------------------------------------
           Crude |   1.986735      1.295122   3.046425
(exact)
    M-H combined |   1.841748      1.217433   2.786219
-------------------------------------------------------------------
Test of homogeneity (M-H)      chi2(1) =     0.72  Pr>chi2 = 0.3969

                   Test that combined OR = 1:
                                Mantel-Haenszel chi2(1) =      8.44
                                                Pr>chi2 =    0.0037

. cc diarrhea clindaquinol
                                                         Proportion
                 |   Exposed   Unexposed  |      Total     Exposed
-----------------+------------------------+------------------------
           Cases |         1         146  |        147       0.0068
        Controls |         2         273  |        275       0.0073
-----------------+------------------------+------------------------
           Total |         3         419  |        422       0.0071
                 |                        |
                 |      Point estimate    |    [95% Conf. Interval]
                 |------------------------+------------------------
      Odds ratio |         .9349315       |    .0157406    18.10196
(exact)
 Prev. frac. ex. |         .0650685       |   -17.10196    .9842594
(exact)
 Prev. frac. pop |         .0004732       |
                 +-------------------------------------------------
                               chi2(1) =     0.00  Pr>chi2 = 0.9563

. cc diarrhea ptzvanco
                                                         Proportion
                 |   Exposed   Unexposed  |      Total     Exposed
-----------------+------------------------+------------------------
           Cases |        43         104  |        147       0.2925
        Controls |        55         220  |        275       0.2000
-----------------+------------------------+------------------------
           Total |        98         324  |        422       0.2322
                 |                        |
                 |      Point estimate    |    [95% Conf. Interval]
                 |------------------------+------------------------
      Odds ratio |         1.653846       |    1.011731    2.690185
(exact)
 Attr. frac. ex. |         .3953488       |    .0115952    .6282783
(exact)
 Attr. frac. pop |         .1156463       |
                 +-------------------------------------------------
```

```
                                 chi2(1) =     4.60  Pr>chi2 = 0.0320

. cc diarrhea ptz,by(vanco)

           vanco |       OR       [95% Conf. Interval]   M-H Weight
-----------------+-------------------------------------------------
               0 |   2.294956      .9483133   5.428606     3.692308
(exact)
               1 |   .9887701      .5190266   1.886508     10.68571
(exact)
-----------------+-------------------------------------------------
           Crude |   1.768147      1.123842   2.773244
(exact)
    M-H combined |   1.324202      .8196156   2.139429
-------------------------------------------------------------------
Test of homogeneity (M-H)      chi2(1) =     2.76  Pr>chi2 = 0.0967

                   Test that combined OR = 1:
                                Mantel-Haenszel chi2(1) =      1.37
                                                Pr>chi2 =    0.2425

. cc diarrhea ptzclinda
                                                         Proportion
                 |   Exposed   Unexposed  |      Total     Exposed
-----------------+------------------------+------------------------
           Cases |         1         146  |        147       0.0068
        Controls |         2         273  |        275       0.0073
-----------------+------------------------+------------------------
           Total |         3         419  |        422       0.0071
                 |                        |
                 |      Point estimate    |    [95% Conf. Interval]
                 |------------------------+------------------------
      Odds ratio |         .9349315       |    .0157406    18.10196
(exact)
 Prev. frac. ex. |         .0650685       |   -17.10196    .9842594
(exact)
 Prev. frac. pop |         .0004732       |
                 +-------------------------------------------------
                                 chi2(1) =     0.00  Pr>chi2 = 0.9563

. cc diarrhea vancoclinda
                                                         Proportion
                 |   Exposed   Unexposed  |      Total     Exposed
-----------------+------------------------+------------------------
           Cases |         4         143  |        147       0.0272
        Controls |         4         271  |        275       0.0145
-----------------+------------------------+------------------------
           Total |         8         414  |        422       0.0190
                 |                        |
                 |      Point estimate    |    [95% Conf. Interval]
                 |------------------------+------------------------
      Odds ratio |         1.895105       |    .3470412    10.31632
(exact)
 Attr. frac. ex. |         .4723247       |   -1.881503    .9030663
(exact)
 Attr. frac. pop |         .0128524       |
                 +-------------------------------------------------
```

```
                           chi2(1) =     0.83  Pr>chi2 = 0.3634

. cc diarrhea cefaquinolptz
                                                         Proportion
                 |   Exposed   Unexposed  |      Total     Exposed
-----------------+------------------------+------------------------
           Cases |         2         145  |        147       0.0136
        Controls |         1         274  |        275       0.0036
-----------------+------------------------+------------------------
           Total |         3         419  |        422       0.0071
                 |                        |
                 |      Point estimate    |    [95% Conf. Interval]
                 |------------------------+------------------------
      Odds ratio |         3.77931        |    .1945774    223.7391
(exact)
 Attr. frac. ex. |        .7354015        |   -4.139343    .9955305
(exact)
 Attr. frac. pop |        .0100055        |
                 +-------------------------------------------------
                           chi2(1) =     1.35  Pr>chi2 = 0.2455

. cc diarrhea cefaquinolvanco
                                                         Proportion
                 |   Exposed   Unexposed  |      Total     Exposed
-----------------+------------------------+------------------------
           Cases |         4         143  |        147       0.0272
        Controls |         2         273  |        275       0.0073
-----------------+------------------------+------------------------
           Total |         6         416  |        422       0.0142
                 |                        |
                 |      Point estimate    |    [95% Conf. Interval]
                 |------------------------+------------------------
      Odds ratio |        3.818182        |    .5380432    42.53738
(exact)
 Attr. frac. ex. |        .7380952        |   -.8585867    .9764913
(exact)
 Attr. frac. pop |        .0200842        |
                 +-------------------------------------------------
                           chi2(1) =     2.72  Pr>chi2 = 0.0993

. cc diarrhea cefaquinolclinda

                 | cefaquinolclinda       |                Proportion
                 |   Exposed   Unexposed  |      Total     Exposed
-----------------+------------------------+------------------------
           Cases |         0         147  |        147       0.0000
        Controls |         1         274  |        275       0.0036
-----------------+------------------------+------------------------
           Total |         1         421  |        422       0.0024
                 |                        |
                 |      Point estimate    |    [95% Conf. Interval]
                 |------------------------+------------------------
      Odds ratio |               0        |           0           .
(Cornfield)
 Prev. frac. ex. |               1        |           .           1
(Cornfield)
 Prev. frac. pop |               .        |
```

```
                 +-------------------------------------------------
                               chi2(1) =     0.54  Pr>chi2 = 0.4642

 Note: Exact confidence levels not possible with zero count cells.

. cc diarrhea cefaptzvanco
                                                         Proportion
                 |   Exposed   Unexposed  |      Total     Exposed
-----------------+------------------------+------------------------
           Cases |        13         134  |        147       0.0884
        Controls |         5         270  |        275       0.0182
-----------------+------------------------+------------------------
           Total |        18         404  |        422       0.0427
                 |                        |
                 |      Point estimate    |    [95% Conf. Interval]
                 |------------------------+------------------------
      Odds ratio |         5.238806       |    1.700323     19.0872
(exact)
 Attr. frac. ex. |         .8091168       |    .4118763    .9476089
(exact)
 Attr. frac. pop |         .0715545       |
                 +-------------------------------------------------
                               chi2(1) =    11.58  Pr>chi2 = 0.0007

. cc diarrhea cefaptz,by(vanco)

           vanco |       OR       [95% Conf. Interval]   M-H Weight
-----------------+-------------------------------------------------
               0 |   1.268116       .021191   24.70076     .5587045
(exact)
               1 |   3.778125      1.181094   14.11106     1.828571
(exact)
-----------------+-------------------------------------------------
           Crude |   4.030075      1.475544   12.04781
(exact)
    M-H combined |   3.190697       1.22231   8.328939
-------------------------------------------------------------------
Test of homogeneity (M-H)      chi2(1) =     0.65  Pr>chi2 = 0.4186

                   Test that combined OR = 1:
                                Mantel-Haenszel chi2(1) =      5.94
                                                Pr>chi2 =    0.0148

. cc diarrhea cefaptzclinda

                 | cefaPTZclinda          |              Proportion
                 |   Exposed   Unexposed  |      Total     Exposed
-----------------+------------------------+------------------------
           Cases |         1         146  |        147       0.0068
        Controls |         0         275  |        275       0.0000
-----------------+------------------------+------------------------
           Total |         1         421  |        422       0.0024
                 |                        |
                 |      Point estimate    |    [95% Conf. Interval]
                 |------------------------+------------------------
      Odds ratio |                .       |           0           .
(Cornfield)
```

```
 Attr. frac. ex. |                1       |          .          .
(Cornfield)
 Attr. frac. pop |         .0068027       |
                 +-------------------------------------------------
                               chi2(1) =     1.88  Pr>chi2 = 0.1709

 Note: Exact confidence levels not possible with zero count cells.

. cc diarrhea cefavancoclinda
                                                         Proportion
                 |   Exposed   Unexposed  |      Total     Exposed
-----------------+------------------------+------------------------
           Cases |         2         145  |        147       0.0136
        Controls |         1         274  |        275       0.0036
-----------------+------------------------+------------------------
           Total |         3         419  |        422       0.0071
                 |                        |
                 |      Point estimate    |    [95% Conf. Interval]
                 |------------------------+------------------------
      Odds ratio |          3.77931       |    .1945774    223.7391
(exact)
 Attr. frac. ex. |         .7354015       |   -4.139343    .9955305
(exact)
 Attr. frac. pop |         .0100055       |
                 +-------------------------------------------------
                               chi2(1) =     1.35  Pr>chi2 = 0.2455

. cc diarrhea quinolptzvanco
                                                         Proportion
                 |   Exposed   Unexposed  |      Total     Exposed
-----------------+------------------------+------------------------
           Cases |        10         137  |        147       0.0680
        Controls |        11         264  |        275       0.0400
-----------------+------------------------+------------------------
           Total |        21         401  |        422       0.0498
                 |                        |
                 |      Point estimate    |    [95% Conf. Interval]
                 |------------------------+------------------------
      Odds ratio |         1.751825       |    .6484366      4.6638
(exact)
 Attr. frac. ex. |         .4291667       |   -.5421708    .7855826
(exact)
 Attr. frac. pop |          .029195       |
                 +-------------------------------------------------
                               chi2(1) =     1.59  Pr>chi2 = 0.2071

. cc diarrhea quinolptzclinda

                 | quinolPTZclinda        |               Proportion
                 |   Exposed   Unexposed  |      Total     Exposed
-----------------+------------------------+------------------------
           Cases |         0         147  |        147       0.0000
        Controls |         0         275  |        275       0.0000
-----------------+------------------------+------------------------
           Total |         0         422  |        422       0.0000
                 |                        |
                 |      Point estimate    |    [95% Conf. Interval]
```

```
                 |------------------------+------------------------
      Odds ratio |                 .      |          .           .
(Cornfield)
 Attr. frac. ex. |                 .      |          .           .
(Cornfield)
 Attr. frac. pop |                 .      |
                 +-------------------------------------------------
                               chi2(1) =        .  Pr>chi2 =      .

 Note: Exact confidence levels not possible with zero count cells.

. cc diarrhea quinolvancoclinda

                 | quinolvancoclinda      |           Proportion
                 |   Exposed   Unexposed  |      Total     Exposed
-----------------+------------------------+------------------------
           Cases |         0         147  |        147       0.0000
        Controls |         1         274  |        275       0.0036
-----------------+------------------------+------------------------
           Total |         1         421  |        422       0.0024
                 |                        |
                 |      Point estimate    |    [95% Conf. Interval]
                 |------------------------+------------------------
      Odds ratio |                 0      |          0           .
(Cornfield)
 Prev. frac. ex. |                 1      |          .           1
(Cornfield)
 Prev. frac. pop |                 .      |
                 +-------------------------------------------------
                               chi2(1) =     0.54  Pr>chi2 = 0.4642

 Note: Exact confidence levels not possible with zero count cells.

. cc diarrhea quinolvancoclinda, exact

                 | quinolvancoclinda      |           Proportion
                 |   Exposed   Unexposed  |      Total     Exposed
-----------------+------------------------+------------------------
           Cases |         0         147  |        147       0.0000
        Controls |         1         274  |        275       0.0036
-----------------+------------------------+------------------------
           Total |         1         421  |        422       0.0024
                 |                        |
                 |      Point estimate    |    [95% Conf. Interval]
                 |------------------------+------------------------
      Odds ratio |                 0      |          0           .
(Cornfield)
 Prev. frac. ex. |                 1      |          .           1
(Cornfield)
 Prev. frac. pop |                 .      |
                 +-------------------------------------------------
                                  1-sided Fisher's exact P = 0.6517
                                  2-sided Fisher's exact P = 1.0000

 Note: Exact confidence levels not possible with zero count cells.

. cc diarrhea ptzvancoclinda
```

```
                                                           Proportion
                 |   Exposed   Unexposed  |      Total      Exposed
-----------------+------------------------+------------------------
           Cases |         1         146  |        147       0.0068
        Controls |         2         273  |        275       0.0073
-----------------+------------------------+------------------------
           Total |         3         419  |        422       0.0071
                 |                        |
                 |      Point estimate    |    [95% Conf. Interval]
                 |------------------------+------------------------
      Odds ratio |         .9349315       |    .0157406    18.10196
(exact)
 Prev. frac. ex. |         .0650685       |   -17.10196    .9842594
(exact)
 Prev. frac. pop |         .0004732       |
                 +-------------------------------------------------
                               chi2(1) =     0.00  Pr>chi2 = 0.9563

. cc diarrhea carbaptzvanco
                                                           Proportion
                 |   Exposed   Unexposed  |      Total      Exposed
-----------------+------------------------+------------------------
           Cases |        13         134  |        147       0.0884
        Controls |         4         271  |        275       0.0145
-----------------+------------------------+------------------------
           Total |        17         405  |        422       0.0403
                 |                        |
                 |      Point estimate    |    [95% Conf. Interval]
                 |------------------------+------------------------
      Odds ratio |         6.572761       |     1.97234    28.07061
(exact)
 Attr. frac. ex. |         .8478569       |    .4929881    .9643756
(exact)
 Attr. frac. pop |         .0749805       |
                 +-------------------------------------------------
                               chi2(1) =    13.53  Pr>chi2 = 0.0002

. cc diarrhea carba,by(ptzvanco)

        PTZvanco |       OR       [95% Conf. Interval]   M-H Weight
-----------------+-------------------------------------------------
               0 |   2.407407      1.451342   4.008432     10.83333
(exact)
               1 |   1.997449      .8092606   4.997472            4
(exact)
-----------------+-------------------------------------------------
           Crude |    2.35977      1.531019   3.647314
(exact)
    M-H combined |   2.296857      1.516951   3.477734
-------------------------------------------------------------------
Test of homogeneity (M-H)      chi2(1) =     0.15  Pr>chi2 = 0.7023

                   Test that combined OR = 1:
                                Mantel-Haenszel chi2(1) =     15.67
                                                Pr>chi2 =    0.0001

. cc diarrhea carbacef
```

```
                                                         Proportion
                 |   Exposed   Unexposed  |      Total     Exposed
-----------------+------------------------+------------------------
           Cases |         7         140  |        147       0.0476
        Controls |         4         271  |        275       0.0145
-----------------+------------------------+------------------------
           Total |        11         411  |        422       0.0261
                 |                        |
                 |      Point estimate    |    [95% Conf. Interval]
                 |------------------------+------------------------
      Odds ratio |          3.3875        |   .8418474     16.0028
(exact)
 Attr. frac. ex. |         .704797        |  -.1878637    .9375109
(exact)
 Attr. frac. pop |        .0335618        |
                 +-------------------------------------------------
                              chi2(1) =     4.13  Pr>chi2 = 0.0422

. cc diarrhea carba,by(cefalosp)

        cefalosp |       OR       [95% Conf. Interval]   M-H Weight
-----------------+-------------------------------------------------
               0 |   2.571272      1.560353   4.257797      10.7929
(exact)
               1 |   1.672727      .6428531   4.385353     3.928571
(exact)
-----------------+-------------------------------------------------
           Crude |    2.35977      1.531019   3.647314
(exact)
    M-H combined |   2.331486      1.539057   3.531922
-------------------------------------------------------------------
Test of homogeneity (M-H)      chi2(1) =     0.72  Pr>chi2 = 0.3952

                   Test that combined OR = 1:
                                Mantel-Haenszel chi2(1) =     16.24
                                                Pr>chi2 =    0.0001

. cc diarrhea carbaquinol
                                                         Proportion
                 |   Exposed   Unexposed  |      Total     Exposed
-----------------+------------------------+------------------------
           Cases |         4         143  |        147       0.0272
        Controls |         1         274  |        275       0.0036
-----------------+------------------------+------------------------
           Total |         5         417  |        422       0.0118
                 |                        |
                 |      Point estimate    |    [95% Conf. Interval]
                 |------------------------+------------------------
      Odds ratio |        7.664336        |   .7458212    378.7311
(exact)
 Attr. frac. ex. |        .8695255        |   -.340804    .9973596
(exact)
 Attr. frac. pop |        .0236606        |
                 +-------------------------------------------------
                              chi2(1) =     4.55  Pr>chi2 = 0.0330

. cc diarrhea carba,by(quinol)
```

```
          quinol |       OR       [95% Conf. Interval]   M-H Weight
-----------------+-------------------------------------------------
               0 |   2.470082      1.547507   3.954032     12.42246
(exact)
               1 |    .907563      .2058394    3.90845     2.479167
(exact)
-----------------+-------------------------------------------------
           Crude |    2.35977      1.531019   3.647314
(exact)
    M-H combined |   2.210127       1.45652   3.353653
-------------------------------------------------------------------
Test of homogeneity (M-H)      chi2(1) =     2.11  Pr>chi2 = 0.1464

                   Test that combined OR = 1:
                                Mantel-Haenszel chi2(1) =     14.16
                                                Pr>chi2 =    0.0002

. cc diarrhea carbaptz
                                                         Proportion
                 |   Exposed   Unexposed  |      Total     Exposed
-----------------+------------------------+------------------------
           Cases |        13         134  |        147       0.0884
        Controls |         4         271  |        275       0.0145
-----------------+------------------------+------------------------
           Total |        17         405  |        422       0.0403
                 |                        |
                 |      Point estimate    |    [95% Conf. Interval]
                 |------------------------+------------------------
      Odds ratio |         6.572761       |     1.97234    28.07061
(exact)
 Attr. frac. ex. |         .8478569       |    .4929881    .9643756
(exact)
 Attr. frac. pop |         .0749805       |
                 +-------------------------------------------------
                               chi2(1) =    13.53  Pr>chi2 = 0.0002

. cc diarrhea carba,by(ptz)

             ptz |       OR       [95% Conf. Interval]   M-H Weight
-----------------+-------------------------------------------------
               0 |   2.846667      1.646312   4.960538     8.644068
(exact)
               1 |   1.652174       .767982   3.567985     5.976378
(exact)
-----------------+-------------------------------------------------
           Crude |    2.35977      1.531019   3.647314
(exact)
    M-H combined |   2.358396      1.555718   3.575218
-------------------------------------------------------------------
Test of homogeneity (M-H)      chi2(1) =     1.48  Pr>chi2 = 0.2241

                   Test that combined OR = 1:
                                Mantel-Haenszel chi2(1) =     16.67
                                                Pr>chi2 =    0.0000

. cc diarrhea carbavanco
```

```
                                                         Proportion
                 |   Exposed   Unexposed  |      Total     Exposed
-----------------+------------------------+------------------------
           Cases |        24         123  |        147      0.1633
        Controls |        14         261  |        275      0.0509
-----------------+------------------------+------------------------
           Total |        38         384  |        422      0.0900
                 |                        |
                 |      Point estimate    |    [95% Conf. Interval]
                 |------------------------+------------------------
      Odds ratio |         3.637631       |    1.732424    7.860149
(exact)
 Attr. frac. ex. |         .7250958       |    .4227742    .8727759
(exact)
 Attr. frac. pop |          .118383       |
                 +-------------------------------------------------
                               chi2(1) =    14.76  Pr>chi2 = 0.0001

. cc diarrhea carba,by(vanco)

           vanco |       OR       [95% Conf. Interval]   M-H Weight
-----------------+-------------------------------------------------
               0 |   2.229102      1.217148   4.075736     7.846154
(exact)
               1 |   1.851596      .9139548   3.809442     6.622857
(exact)
-----------------+-------------------------------------------------
           Crude |    2.35977      1.531019   3.647314
(exact)
    M-H combined |   2.056307      1.338079   3.160052
-------------------------------------------------------------------
Test of homogeneity (M-H)      chi2(1) =     0.18  Pr>chi2 = 0.6753

                   Test that combined OR = 1:
                                Mantel-Haenszel chi2(1) =     10.99
                                                Pr>chi2 =    0.0009

. cc diarrhea carbaclinda
                                                         Proportion
                 |   Exposed   Unexposed  |      Total     Exposed
-----------------+------------------------+------------------------
           Cases |         2         145  |        147      0.0136
        Controls |         1         274  |        275      0.0036
-----------------+------------------------+------------------------
           Total |         3         419  |        422      0.0071
                 |                        |
                 |      Point estimate    |    [95% Conf. Interval]
                 |------------------------+------------------------
      Odds ratio |          3.77931       |    .1945774    223.7391
(exact)
 Attr. frac. ex. |         .7354015       |   -4.139343    .9955305
(exact)
 Attr. frac. pop |         .0100055       |
                 +-------------------------------------------------
                               chi2(1) =     1.35  Pr>chi2 = 0.2455

. cc diarrhea carbacefaquinol
```

```
                 | carbacefaquinol        |           Proportion
                 |   Exposed   Unexposed  |      Total     Exposed
-----------------+------------------------+------------------------
           Cases |         2         145  |        147       0.0136
        Controls |         0         275  |        275       0.0000
-----------------+------------------------+------------------------
           Total |         2         420  |        422       0.0047
                 |                        |
                 |      Point estimate    |    [95% Conf. Interval]
                 |------------------------+------------------------
      Odds ratio |                .       |    .9791263           .
(Cornfield)
 Attr. frac. ex. |                1       |   -.0213187           .
(Cornfield)
 Attr. frac. pop |         .0136054       |
                 +-------------------------------------------------
                              chi2(1) =     3.76  Pr>chi2 = 0.0525

 Note: Exact confidence levels not possible with zero count cells.

. cc diarrhea carba,by(cefaquinol)

      cefaquinol |       OR       [95% Conf. Interval]   M-H Weight
-----------------+-------------------------------------------------
               0 |   2.436207      1.566891   3.799859     14.04358
(exact)
               1 |          0             0   3.996121     .6666667
(exact)
-----------------+-------------------------------------------------
           Crude |    2.35977      1.531019   3.647314
(exact)
    M-H combined |   2.325798      1.535067   3.523845
-------------------------------------------------------------------
Test of homogeneity (Tarone)   chi2(1) =     2.60  Pr>chi2 = 0.1069

                   Test that combined OR = 1:
                                Mantel-Haenszel chi2(1) =     16.13
                                                Pr>chi2 =    0.0001

. cc diarrhea carbacefaptz
                                                         Proportion
                 |   Exposed   Unexposed  |      Total     Exposed
-----------------+------------------------+------------------------
           Cases |         4         143  |        147       0.0272
        Controls |         2         273  |        275       0.0073
-----------------+------------------------+------------------------
           Total |         6         416  |        422       0.0142
                 |                        |
                 |      Point estimate    |    [95% Conf. Interval]
                 |------------------------+------------------------
      Odds ratio |         3.818182       |    .5380432    42.53738
(exact)
 Attr. frac. ex. |         .7380952       |   -.8585867    .9764913
(exact)
 Attr. frac. pop |         .0200842       |
                 +-------------------------------------------------
```

```
                             chi2(1) =     2.72  Pr>chi2 = 0.0993

. cc diarrhea carbacefavanco
                                                         Proportion
                 |   Exposed   Unexposed  |      Total     Exposed
-----------------+------------------------+------------------------
           Cases |         6         141  |        147       0.0408
        Controls |         4         271  |        275       0.0145
-----------------+------------------------+------------------------
           Total |        10         412  |        422       0.0237
                 |                        |
                 |      Point estimate    |    [95% Conf. Interval]
                 |------------------------+------------------------
      Odds ratio |         2.882979       |    .6693936     14.0851
(exact)
 Attr. frac. ex. |         .6531365       |   -.4938893     .929003
(exact)
 Attr. frac. pop |         .0266586       |
                 +-------------------------------------------------
                             chi2(1) =     2.86  Pr>chi2 = 0.0909

. cc diarrhea carbacefaclinda

                 | carbacefaclinda        |             Proportion
                 |   Exposed   Unexposed  |      Total     Exposed
-----------------+------------------------+------------------------
           Cases |         1         146  |        147       0.0068
        Controls |         0         275  |        275       0.0000
-----------------+------------------------+------------------------
           Total |         1         421  |        422       0.0024
                 |                        |
                 |      Point estimate    |    [95% Conf. Interval]
                 |------------------------+------------------------
      Odds ratio |                .       |           0           .
(Cornfield)
 Attr. frac. ex. |                1       |           .           .
(Cornfield)
 Attr. frac. pop |         .0068027       |
                 +-------------------------------------------------
                             chi2(1) =     1.88  Pr>chi2 = 0.1709

 Note: Exact confidence levels not possible with zero count cells.

. cc diarrhea carbaquinolptz

                 | carbaquinolPTZ         |             Proportion
                 |   Exposed   Unexposed  |      Total     Exposed
-----------------+------------------------+------------------------
           Cases |         1         146  |        147       0.0068
        Controls |         0         275  |        275       0.0000
-----------------+------------------------+------------------------
           Total |         1         421  |        422       0.0024
                 |                        |
                 |      Point estimate    |    [95% Conf. Interval]
                 |------------------------+------------------------
      Odds ratio |                .       |           0           .
(Cornfield)
```

```
 Attr. frac. ex. |                1       |          .          .
(Cornfield)
 Attr. frac. pop |         .0068027       |
                 +-------------------------------------------------
                               chi2(1) =     1.88  Pr>chi2 = 0.1709

 Note: Exact confidence levels not possible with zero count cells.

. cc diarrhea carbaquinolvanco

                 | carbaquinolvanco       |           Proportion
                 |   Exposed   Unexposed  |      Total     Exposed
-----------------+------------------------+------------------------
           Cases |         3         144  |        147       0.0204
        Controls |         0         275  |        275       0.0000
-----------------+------------------------+------------------------
           Total |         3         419  |        422       0.0071
                 |                        |
                 |      Point estimate    |    [95% Conf. Interval]
                 |------------------------+------------------------
      Odds ratio |                .       |     1.47666           .
(Cornfield)
 Attr. frac. ex. |                1       |    .3227962           .
(Cornfield)
 Attr. frac. pop |         .0204082       |
                 +-------------------------------------------------
                               chi2(1) =     5.65  Pr>chi2 = 0.0174

 Note: Exact confidence levels not possible with zero count cells.

. cc diarrhea carba,by(quinolvanco)

    quinolvanc~a |       OR       [95% Conf. Interval]   M-H Weight
-----------------+-------------------------------------------------
               0 |   2.379939      1.543513   3.679788     14.72922
(exact)
               1 |          .             .          .            0
(exact)
-----------------+-------------------------------------------------
           Crude |    2.35977      1.531019   3.647314
(exact)
    M-H combined |   2.379939      1.574565   3.597252
-------------------------------------------------------------------
Test of homogeneity (Tarone)   chi2(1) =     0.00  Pr>chi2 = 1.0000

                   Test that combined OR = 1:
                                Mantel-Haenszel chi2(1) =         .
                                                Pr>chi2 =         .

. cc diarrhea carbaquinolclinda

                 | carbaquinolclinda      |           Proportion
                 |   Exposed   Unexposed  |      Total     Exposed
-----------------+------------------------+------------------------
           Cases |         0         147  |        147       0.0000
        Controls |         1         274  |        275       0.0036
-----------------+------------------------+------------------------
```

```
           Total |         1          421  |        422       0.0024
                 |                         |
                 |      Point estimate     |    [95% Conf. Interval]
                 |------------------------+------------------------
      Odds ratio |                0        |         0           .
(Cornfield)
 Prev. frac. ex. |                1        |         .           1
(Cornfield)
 Prev. frac. pop |                .        |
                 +-------------------------------------------------
                               chi2(1) =     0.54  Pr>chi2 = 0.4642

 Note: Exact confidence levels not possible with zero count cells.

. cc diarrhea carbaptzclinda

                 | carbaPTZclinda          |             Proportion
                 |   Exposed   Unexposed   |      Total     Exposed
-----------------+------------------------+------------------------
           Cases |         1          146  |        147       0.0068
        Controls |         0          275  |        275       0.0000
-----------------+------------------------+------------------------
           Total |         1          421  |        422       0.0024
                 |                         |
                 |      Point estimate     |    [95% Conf. Interval]
                 |------------------------+------------------------
      Odds ratio |                .        |         0           .
(Cornfield)
 Attr. frac. ex. |                1        |         .           .
(Cornfield)
 Attr. frac. pop |         .0068027        |
                 +-------------------------------------------------
                               chi2(1) =     1.88  Pr>chi2 = 0.1709

 Note: Exact confidence levels not possible with zero count cells.

. cc diarrhea carbavancoclinda

                 | carbavancoclinda        |             Proportion
                 |   Exposed   Unexposed   |      Total     Exposed
-----------------+------------------------+------------------------
           Cases |         2          145  |        147       0.0136
        Controls |         0          275  |        275       0.0000
-----------------+------------------------+------------------------
           Total |         2          420  |        422       0.0047
                 |                         |
                 |      Point estimate     |    [95% Conf. Interval]
                 |------------------------+------------------------
      Odds ratio |                .        |    .9791263           .
(Cornfield)
 Attr. frac. ex. |                1        |   -.0213187           .
(Cornfield)
 Attr. frac. pop |         .0136054        |
                 +-------------------------------------------------
                               chi2(1) =     3.76  Pr>chi2 = 0.0525

 Note: Exact confidence levels not possible with zero count cells.
```

```
. cc diarrhea carba,by(vancoclinda)

     vancoclinda |       OR       [95% Conf. Interval]   M-H Weight
-----------------+-------------------------------------------------
               0 |   2.410088      1.555596   3.744238     14.31884
(exact)
               1 |          0             0          .           .5
(exact)
-----------------+-------------------------------------------------
           Crude |    2.35977      1.531019   3.647314
(exact)
    M-H combined |   2.328769      1.539716   3.522186
-------------------------------------------------------------------
Test of homogeneity (Tarone)   chi2(1) =     2.39  Pr>chi2 = 0.1219

                   Test that combined OR = 1:
                                Mantel-Haenszel chi2(1) =     16.36
                                                Pr>chi2 =    0.0001

.
```

Simplified model-building strategy.

```
. use "/Users/ericochoahein/Desktop/stata database.dta"

. sw logistic diarrhea feb_neutr leukemia lymphoma surgery intraabdsepsis
nongastr_inf
>  kidney_tr autoimm chemotx ppi ent_nut atb quinol cipro cipro_ppi
meroimi erta carba
>  vanco ptz ceftria cefta cefalosp3rd hosp hosp_other hosp_both, pe(0.1)
between-term collinearity, variable cefta
r(498);
```

We begun with forward stepwise selection of variables with p<0.1 in bivariate analysis. Ceftazidime was dropped due to collinearity.

```
. sw logistic diarrhea feb_neutr leukemia lymphoma surgery intraabdsepsis
nongastr_inf
>  kidney_tr autoimm chemotx ppi ent_nut atb quinol cipro cipro_ppi
meroimi erta carba
>  vanco ptz ceftria cefalosp3rd hosp hosp_other hosp_both, pe(0.1)
                      begin with empty model
p = 0.0000 <  0.1000  adding   atb
p = 0.0099 <  0.1000  adding   cipro_ppi
p = 0.0218 <  0.1000  adding   feb_neutr
p = 0.0408 <  0.1000  adding   intraabdsepsis
p = 0.0549 <  0.1000  adding   hosp_other
p = 0.0333 <  0.1000  adding   hosp_both

Logistic regression                                Number of obs     =
347
                                                   LR chi2(6)        =
60.20
                                                   Prob > chi2       =
0.0000
Log likelihood = -193.65514                        Pseudo R2         =
0.1345

------------------------------------------------------------------------
-------
      diarrhea | Odds Ratio   Std. Err.      z    P>|z|     [95% Conf.
Interval]
---------------+--------------------------------------------------------
-------
           atb |   1.292953   .0876515     3.79   0.000     1.132083
1.476683
     cipro_ppi |   7.610395    6.10682     2.53   0.011     1.578979
36.68073
     feb_neutr |   4.599743    2.84653     2.47   0.014     1.367638
15.4702
intraabdsepsis |   2.185786   .8834073     1.93   0.053     .9898879
4.826466
    hosp_other |   2.216023   .8219459     2.15   0.032     1.071161
4.584519
     hosp_both |   2.652325   1.215811     2.13   0.033     1.080042
6.513476
```

```
         _cons |    .179915   .0404358    -7.63   0.000     .1158138
.2794952
--------------------------------------------------------------------------
-------
```

```
. sw logistic diarrhea atb cipro_ppi feb_neutr intraabdsepsis hosp_other
hosp_both, pr(0.05)
                      begin with full model
p = 0.0734 >= 0.0500  removing intraabdsepsis

Logistic regression                               Number of obs     =
360
                                                  LR chi2(5)        =
57.88
                                                  Prob > chi2       =
0.0000
Log likelihood = -202.87674                       Pseudo R2         =
0.1248

--------------------------------------------------------------------------
-----
    diarrhea | Odds Ratio   Std. Err.      z    P>|z|     [95% Conf.
Interval]
-------------+------------------------------------------------------------
-----
         atb |    1.31325   .0852334     4.20   0.000     1.156384
1.491394
   cipro_ppi |   8.418052   6.664293     2.69   0.007     1.783789
39.72644
   feb_neutr |   4.236586   2.609464     2.34   0.019     1.266866
14.16776
   hosp_both |   2.719325   1.231027     2.21   0.027     1.119762
6.60384
  hosp_other |   2.212331   .7996409     2.20   0.028     1.089396
4.492774
       _cons |   .1854695   .0407676    -7.67   0.000     .1205513
.2853469
--------------------------------------------------------------------------
-----
```

Backward stepwise regression using p<0.05 as cutoff value removed intraabdominal infection from the model.

```
. logistic diarrhea atb cipro_ppi feb_neutr hosp_both

Logistic regression                               Number of obs     =
360
                                                  LR chi2(4)        =
53.10
                                                  Prob > chi2       =
0.0000
Log likelihood = -205.26683                       Pseudo R2         =
0.1145
```

```
------------------------------------------------------------------------------
    diarrhea | Odds Ratio   Std. Err.      z    P>|z|     [95% Conf. Interval]
-------------+----------------------------------------------------------------
         atb |   1.340327   .0859937     4.57   0.000     1.181949    1.519927
   cipro_ppi |   8.154215   6.437685     2.66   0.008     1.735264    38.31763
   feb_neutr |   4.142944   2.532367     2.33   0.020     1.250298    13.72791
   hosp_both |   2.439582   1.099823     1.98   0.048     1.008261    5.902797
       _cons |   .1962397   .0424662    -7.53   0.000     .1284066    .2999071
------------------------------------------------------------------------------

. logistic diarrhea atb cipro_ppi feb_neutr hosp_other

Logistic regression                               Number of obs     =        360
                                                  LR chi2(4)        =      53.08
                                                  Prob > chi2       =     0.0000
Log likelihood = -205.27799                       Pseudo R2         =     0.1145

------------------------------------------------------------------------------
    diarrhea | Odds Ratio   Std. Err.      z    P>|z|     [95% Conf. Interval]
-------------+----------------------------------------------------------------
         atb |    1.31658   .0847406     4.27   0.000     1.160541    1.493599
   cipro_ppi |   7.752613   6.126248     2.59   0.010     1.647457     36.4823
   feb_neutr |   4.227432   2.599284     2.34   0.019     1.266793    14.10742
  hosp_other |     2.0255   .7264441     1.97   0.049     1.002881    4.090864
       _cons |   .2012113   .0429341    -7.51   0.000     .1324416    .3056893
------------------------------------------------------------------------------
```

Hospitalization in another hospital and hospitalization in both an external and our hospital provide similar information to the model. Pseudo R2 does not change. We decided to include hospitalization in another hospital because hospitalization in our hospital only was not significant in bivariate analysis and hospitalization in both an external hospital and ours is skewed by hospitalization in an external hospital.

```
. logistic diarrhea atb cipro_ppi feb_neutr hosp_other intraabdsepsis

Logistic regression                              Number of obs     =
360
                                                 LR chi2(5)        =
56.48
                                                 Prob > chi2       =
0.0000
Log likelihood = -203.57657                      Pseudo R2         =
0.1218

--------------------------------------------------------------------------
-------
      diarrhea | Odds Ratio   Std. Err.      z    P>|z|     [95% Conf.
Interval]
---------------+----------------------------------------------------------
-------
           atb |   1.288474   .0845166     3.86   0.000     1.133031
1.465243
     cipro_ppi |   8.074547   6.398299     2.64   0.008     1.708532
38.16043
     feb_neutr |   4.612262   2.846802     2.48   0.013     1.375724
15.4631
    hosp_other |   1.994951   .7215496     1.91   0.056     .9818903
4.053233
intraabdsepsis |   2.068498   .8128299     1.85   0.064     .9575688
4.46828
         _cons |   .1969019   .0423471    -7.56   0.000     .1291768
.3001342
--------------------------------------------------------------------------
-------
```

Introduction of intraabdmoninal infection improves the model significantly, as judged by the change in the pseudo R2 value(that was significant). Nonetheless, two variables take on p values higher than 0.05 (although lower than our prespecified value of 0.1).

```
. corr diarrhea atb cipro_ppi feb_neutr hosp_other intraabdsepsis
(obs=360)

             | diarrhea      atb cipro~pi feb_ne~r hosp_o~r intraa~s
-------------+------------------------------------------------------
    diarrhea |   1.0000
         atb |   0.2980   1.0000
   cipro_ppi |   0.2292   0.2018   1.0000
   feb_neutr |   0.1841   0.1213   0.1574   1.0000
  hosp_other |   0.1450   0.1601   0.0128   0.0075   1.0000
intraabdse~s |   0.1457   0.2060   0.0277  -0.0236   0.0636   1.0000


. corr diarrhea atb cipro_ppi feb_neutr hosp_other hosp_both
(obs=360)

             | diarrhea      atb cipro~pi feb_ne~r hosp_o~r hosp_b~h
-------------+------------------------------------------------------
    diarrhea |   1.0000
```

```
         atb |   0.2980   1.0000
   cipro_ppi |   0.2292   0.2018   1.0000
   feb_neutr |   0.1841   0.1213   0.1574   1.0000
  hosp_other |   0.1450   0.1601   0.0128   0.0075   1.0000
   hosp_both |   0.0975   0.0220  -0.0545  -0.0012  -0.0937   1.0000
```

Diarrhea is better correlated to intraabdominal infection than hospitalization in both an external and our hospital.

```
. logistic diarrhea atb cipro_ppi feb_neutr hosp_other intraabdsepsis

Logistic regression                               Number of obs     =
360
                                                  LR chi2(5)        =
56.48
                                                  Prob > chi2       =
0.0000
Log likelihood = -203.57657                       Pseudo R2         =
0.1218

--------------------------------------------------------------------------
-------
      diarrhea | Odds Ratio   Std. Err.      z    P>|z|     [95% Conf.
Interval]
---------------+----------------------------------------------------------
-------
           atb |   1.288474   .0845166     3.86   0.000     1.133031
1.465243
     cipro_ppi |   8.074547   6.398299     2.64   0.008     1.708532
38.16043
     feb_neutr |   4.612262   2.846802     2.48   0.013     1.375724
15.4631
    hosp_other |   1.994951   .7215496     1.91   0.056     .9818903
4.053233
intraabdsepsis |   2.068498   .8128299     1.85   0.064     .9575688
4.46828
         _cons |   .1969019   .0423471    -7.56   0.000     .1291768
.3001342
--------------------------------------------------------------------------
-------

. lroc

Logistic model for diarrhea

number of observations =      360
area under ROC curve   =   0.7259

. logistic diarrhea atb cipro_ppi feb_neutr hosp_other hosp_both

Logistic regression                               Number of obs     =
360
                                                  LR chi2(5)        =
57.88
                                                  Prob > chi2       =
0.0000
```

```
Log likelihood = -202.87674                     Pseudo R2         =
0.1248

-------------------------------------------------------------------------
-----
    diarrhea | Odds Ratio   Std. Err.      z    P>|z|     [95% Conf.
Interval]
-------------+-----------------------------------------------------------
-----
         atb |    1.31325   .0852334     4.20   0.000     1.156384
1.491394
   cipro_ppi |   8.418052   6.664293     2.69   0.007     1.783789
39.72644
   feb_neutr |   4.236586   2.609464     2.34   0.019     1.266866
14.16776
  hosp_other |   2.212331   .7996409     2.20   0.028     1.089396
4.492774
   hosp_both |   2.719325   1.231027     2.21   0.027     1.119762
6.60384
       _cons |   .1854695   .0407676    -7.67   0.000     .1205513
.2853469
-------------------------------------------------------------------------
-----

. lroc

Logistic model for diarrhea

number of observations =      360
area under ROC curve   =   0.7275
```

Both models have similar area under the curve values. Although confidence intervals for variables in the latter model do not cross the unit, adjusted odds ratio values do not differ substantially between both models.

```
. logistic diarrhea atb cipro_ppi feb_neutr hosp_other intraabdsepsis

Logistic regression                             Number of obs     =
360
                                                LR chi2(5)        =
56.48
                                                Prob > chi2       =
0.0000
Log likelihood = -203.57657                     Pseudo R2         =
0.1218

-------------------------------------------------------------------------
-------
      diarrhea | Odds Ratio   Std. Err.      z    P>|z|     [95% Conf.
Interval]
---------------+---------------------------------------------------------
-------
           atb |   1.288474   .0845166     3.86   0.000     1.133031
1.465243
```

```
     cipro_ppi |   8.074547   6.398299     2.64   0.008     1.708532
38.16043
     feb_neutr |   4.612262   2.846802     2.48   0.013     1.375724
15.4631
    hosp_other |   1.994951   .7215496     1.91   0.056     .9818903
4.053233
intraabdsepsis |   2.068498   .8128299     1.85   0.064     .9575688
4.46828
         _cons |   .1969019   .0423471    -7.56   0.000     .1291768
.3001342
------------------------------------------------------------------------
-------


. lstat

Logistic model for diarrhea

              -------- True --------
Classified |         D            ~D  |      Total
-----------+--------------------------+-----------
     +     |        43            23  |         66
     -     |        81           213  |        294
-----------+--------------------------+-----------
   Total   |       124           236  |        360

Classified + if predicted Pr(D) >= .5
True D defined as diarrhea != 0
--------------------------------------------------
Sensitivity                     Pr( +| D)   34.68%
Specificity                     Pr( -|~D)   90.25%
Positive predictive value       Pr( D| +)   65.15%
Negative predictive value       Pr(~D| -)   72.45%
--------------------------------------------------
False + rate for true ~D        Pr( +|~D)    9.75%
False - rate for true D         Pr( -| D)   65.32%
False + rate for classified +   Pr(~D| +)   34.85%
False - rate for classified -   Pr( D| -)   27.55%
--------------------------------------------------
Correctly classified                        71.11%
--------------------------------------------------

. logistic diarrhea atb cipro_ppi feb_neutr hosp_other hosp_both

Logistic regression                               Number of obs     =
360
                                                  LR chi2(5)        =
57.88
                                                  Prob > chi2       =
0.0000
Log likelihood = -202.87674                       Pseudo R2         =
0.1248

------------------------------------------------------------------------
-----
    diarrhea | Odds Ratio   Std. Err.      z    P>|z|     [95% Conf.
Interval]
```

```
-------------+----------------------------------------------------------------
-----
         atb |    1.31325   .0852334     4.20   0.000     1.156384
1.491394
   cipro_ppi |   8.418052   6.664293     2.69   0.007     1.783789
39.72644
   feb_neutr |   4.236586   2.609464     2.34   0.019     1.266866
14.16776
  hosp_other |   2.212331   .7996409     2.20   0.028     1.089396
4.492774
   hosp_both |   2.719325   1.231027     2.21   0.027     1.119762
6.60384
       _cons |   .1854695   .0407676    -7.67   0.000     .1205513
.2853469
------------------------------------------------------------------------------
-----

. lstat

Logistic model for diarrhea

              -------- True --------
Classified |         D            ~D  |      Total
-----------+--------------------------+-----------
     +     |        45            20  |         65
     -     |        79           216  |        295
-----------+--------------------------+-----------
   Total   |       124           236  |        360

Classified + if predicted Pr(D) >= .5
True D defined as diarrhea != 0
--------------------------------------------------
Sensitivity                     Pr( +| D)   36.29%
Specificity                     Pr( -|~D)   91.53%
Positive predictive value       Pr( D| +)   69.23%
Negative predictive value       Pr(~D| -)   73.22%
--------------------------------------------------
False + rate for true ~D        Pr( +|~D)    8.47%
False - rate for true D         Pr( -| D)   63.71%
False + rate for classified +   Pr(~D| +)   30.77%
False - rate for classified -   Pr( D| -)   26.78%
--------------------------------------------------
Correctly classified                        72.50%
--------------------------------------------------

.
```

Both models correctly classify cases and controls with similar values.
