# Supplementary material for: Factors associated with an outbreak of hospital-onset, healthcare facility-associated Clostridium difficile infection (HO-HCFA CDI) in a Mexican tertiary care hospital: A case-control study
Source: PLoS One. 2018 May 29;13(5):e0198212. doi: 10.1371/journal.pone.0198212 (PMC5973614; doi:10.1371/journal.pone.0198212)
Supplement: S4 File — (PDF) [file pone.0198212.s004.pdf]

**2017 audit of consumption of hand hygiene products.**

a) *Chlorhexidine soap:*

| Floor | Total liters, 2015 | Total liters, 2016 |
| --- | --- | --- |
| **1** | 1211.8 | 1156.0 |
| **2** | 1416.1 | 1302.6 |
| **3** | 795.5 | 748.2 |
| **4** | 167.4 | 189.2 |
| **ICU** | 831.5 | 654.6 |
| **ER** | 1135.2 | 1132.3 |
| **TOTAL** | 5557.5 | 5182.9 |

b) *Alcohol hand rub:*

| Floor | Total liters, 2015 | Total liters, 2016 |
| --- | --- | --- |
| **1** | 611.1 | 957.7 |
| **2** | 375.8 | 731.1 |
| **3** | 399.4 | 569.9 |
| **4** | 95.0 | 140.0 |
| **ICU** | 246.9 | 385.2 |
| **ER** | 509.1 | 832.3 |
| **TOTAL** | 2237.3 | 3616.2 |
